# The effect of nurse-led interventions in self-efficacy and psychosocial adjustment of individuals with bowel ostomy: a systematic review

**DOI:** 10.1177/17449871261462845

**Published:** 2026-08-20

**Authors:** Joana Portela, Graça Melo, Tânia Mendes, Helga Rafael Henriques

**Affiliations:** Universidade de Lisboa, Escola Superior de Enfermagem, Lisbon, Portugal; Universidade de Lisboa, Innovation and Development Centre of Lisbon (CIDNUR), Lisbon, Portugal; Universidade de Lisboa, Escola Superior de Enfermagem, Lisbon, Portugal; Universidade de Lisboa, Innovation and Development Centre of Lisbon (CIDNUR), Nursing School of Lisbon, Lisbon, Portugal; Registered Nurse at Centre for Training, Research and Clinical Epidemiology at ULSA, Setubal, Portugal; Universidade de Lisboa, Escola Superior de Enfermagem, Lisbon, Portugal; Universidade de Lisboa, Innovation and Development Centre of Lisbon (CIDNUR), Nursing School of Lisbon, Lisbon, Portugal

**Keywords:** bowel ostomy, nurse-led interventions, psychosocial adjustment, self-efficacy, systematic review

## Abstract

**Background:**

Bowel ostomies pose major physical and psychosocial challenges. Self-efficacy supports ostomy self-care, and nurses play a central role through education, training and ongoing support.

**Aim:**

To synthesise evidence on nurse-led interventions that enhance self-efficacy and psychosocial adjustment in adults with bowel ostomy and to identify intervention components and delivery characteristics.

**Methods:**

A systematic review followed the Joanna Briggs Institute (JBI) methodology and the Preferred Reporting Items for Systematic Reviews and Meta-Analyses (PRISMA) 2020 guidelines. Searches were undertaken in five databases (CINAHL, PubMed, Scopus, Web of Science and Cochrane Library) and grey literature (December 2024). Two reviewers independently extracted and appraised data using JBI tools, with results narratively synthesised.

**Results:**

Fourteen studies were included. Nurse-led, multicomponent interventions combining education, training, follow-up and psychosocial support were most effective. Hybrid delivery and post-discharge follow-up strengthened outcomes. Most programmes were unidisciplinary, whereas multidisciplinary approaches provided broader psychosocial benefits. Heterogeneity across studies limited quantitative synthesis.

**Conclusion:**

Nurse-led, multicomponent interventions enhance self-efficacy and psychosocial adjustment, particularly when hybrid, sustained and informed by the theoretical domains framework (TDF). However, the evidence is limited by small sample sizes, heterogeneity of interventions and risk of bias in some studies. Policies should embed these interventions into standard care, emphasising continuity and digital inclusion.

## Background

Epidemiological projections suggest that approximately 1 person per 1000 inhabitants may be living with an ostomy in developed healthcare systems, consistent with estimates from the International Ostomy Association (Paczek et al., 2024). Globally, it is estimated that around 2 million people are living with a stoma, including approximately 650,000 in Europe (Marcomini et al., 2024).

Data from well-characterised healthcare systems further illustrate the magnitude of this condition. For instance, in the United States, between 725,000 and 1 million individuals are estimated to live with an ostomy, with approximately 100,000 new surgeries performed annually (Burgess-Stocks et al., 2022). These figures underscore the significant and growing public health relevance of ostomy care.

Bowel ostomy, including colostomies and ileostomies, are common procedures for conditions such as colorectal cancer, Crohn’s disease, ulcerative colitis, bowel perforations and intestinal obstructions (Bowles et al., 2022; Hansen et al., 2022; Qureshi et al., 2018). Although life-saving and essential to restore physiological function, they bring substantial physical, psychological and social challenges that affect well-being and everyday functioning (Arolfo et al., 2018; Herlufsen and Brødsgaard, 2017; Jayarajah et al., 2016; Vonk-Klaassen et al., 2016).

Living with a stoma demands continuous adaptation (Roy, 1984; Xavier et al., 2024), dependent on self-care behaviours (Lorig et al., 2001; Orem, 2001) and psychosocial adjustments, with self-efficacy as a key factor (Bandura, 1997; Goodman et al., 2022). Individuals must acquire skills and knowledge to manage the appliance, monitor stoma function and peristomal skin integrity and incorporate new routines into daily lives. International guidelines emphasise that structured selfcare interventions in ostomy management, including education, monitoring stoma and peristomal skin, prevent complications through access to professional support (Chabal et al., 2021; Registered Nurses Association of Ontario, 2019; Wound Ostomy and continence Society, 2018). Yet, self-care difficulties remain a major barrier, with even long-term survivors reporting leakage, skin irritation and appliance management problems (Bulkley et al., 2018). Beyond technical care, people often face body image changes, reduced sexual confidence, anxiety about leakage or odour and social withdrawal (Herlufsen and Brødsgaard, 2017; Mota et al., 2015; Thorpe et al., 2014; Vonk-Klaassen et al., 2016). These challenges generate distress and hinder psychosocial adjustment and self-efficacy, compromising the transition to stable, autonomous living (Bozkul et al., 2024).

For individuals with an ostomy, self-care actions extend beyond practical skills: it requires understanding of ostomy function, principles of skin health and dietary management (Bulkley et al., 2018; Liu et al., 2024). This knowledge supports tasks such as changing devices, monitoring complications and integrating care into daily routines (Pinto et al., 2022). Alongside these skills, self-efficacy, empowerment, autonomy and psychosocial adjustment are crucial to coping, reducing complications and improving quality of life (Bozkul et al., 2024). Psychological reactions (frustrations and helplessness), emotional factors, illness beliefs, stigma and health representations also influence acceptance and adherence to behaviours that prevent complications and sustain well-being (Jin et al., 2020; Kittscha et al., 2022; Lim et al., 2015).

Self-efficacy, the belief in one’s ability to perform the required behaviours (Bandura, 1997), in ostomy care reflects confidence in managing tasks, addressing complications and sustaining autonomy (Bozkul et al., 2024). Psychosocial adjustment encompasses how individuals emotionally and socially integrate the stoma into their identity, relationships and life routines (Simmons et al., 2009). Higher self-efficacy and psychosocial adjustment correlate with better self-care, fewer complications and improved quality of life (Bozkul et al., 2024; Clark et al., 2023; Goodman et al., 2022; Mota et al., 2015). Beyond ostomy care, self-efficacy plays a central role in enabling self-management in other chronic conditions. For example, a randomised controlled trial in patients with acute coronary syndrome demonstrated that nurse-led counselling and education enhanced cardiac self-efficacy and supported recovery compared to usual care (Bagheri et al., 2022). These cross-condition findings reinforce the relevance of nurse-led interventions to strengthen self-efficacy in chronic disease contexts.

Ostomy self-care and psychosocial adjustment are domains in which nurses have a central and distinctive role. International guidelines consistently position ostomy nurses as key professionals responsible for pre- and postoperative education, technical training, complication management, behavioural support and ongoing follow-up (Registered Nurses’ Association of Ontario [RNAO], 2019; Wound, Ostomy and Continence Nurses Society [WOCN], 2018; World Council of Enterostomal Therapists [WCET], 2021). From a behavioural perspective, self-care, self-efficacy and transition processes are nursing-led concepts grounded in person-centred care and therapeutic education frameworks (Bandura, 1997; Meleis, 2010; Orem, 2001). Moreover, the transition from hospital to home is predominantly coordinated by nurses, who monitor adaptation and provide continuity of care.

Nurse-led interventions, which typically involve education, skills training, emotional support and follow-up, have been shown to improve both clinical and psychosocial outcomes (Chabal et al., 2021; Registered Nurses Association of Ontario, 2019). They build competencies, promote autonomy and empowerment and strengthen therapeutic relationships that sustain self-management. However, variability in content, structure, duration and delivery of nurse-led programmes has been reported (Bozkul et al., 2024; Goodman et al., 2022; Soares-Pinto et al., 2022), indicating the need for a coherent synthesis to inform evidence-based practice.

This variability highlights the need for a clear evidence synthesis to guide clinical practice. Although past reviews assessed nursing care on general outcomes such as quality of life, a recent systematic review (Bozkul et al., 2024) and a meta-analysis (Goodman et al., 2022) have confirmed that interventions can improve self-efficacy. Despite this, significant gaps remain. Firstly, psychosocial adjustment, a distinct but equally critical outcome, was not explored in depth. Secondly, analysis of the components of nurse-led interventions (the ‘active ingredients’) was insufficient. Finally, subgroup analyses, such as the differential in emergency versus elective surgical contexts, were not performed. This lack of detail limits the translation of findings into tailored, evidence-based clinical practice.

This review aims to address these gaps by evaluating the effect of nurse-led interventions in improving self-efficacy and psychosocial adjustment in bowel ostomy patients, while analysing the core interventions and effects across clinical contexts.

Specifically, we sought to address the following primary (Population, Intervention, Outcome) research question:

- What are the effects of nurse-led interventions (I) targeting self-efficacy and psychosocial adjustment (O) in individuals with a bowel ostomy (P), compared to usual care or other interventions?

We also aim to address the following secondary questions:

1. What is the effect of nurse-led intervention on promoting self-efficacy behaviours and psychosocial adjustment?
2. Is the effect influenced by the surgical condition associated with the formation of the ostomy?
3. Is the effect of interventions influenced by the delivered mode (face-to-face, digital/remote or hybrid interventions)?
4. Is the effect influenced by the timing at which they are delivered (preoperative, transitional care or follow-up)?
5. Is the effect influenced by the professionals involved (unidisciplinary or multidisciplinary)?

By synthesising quantitative studies with comparison groups and validated outcomes, this review seeks to contribute robust evidence to guide clinical practice, inform policy and support the design of future nursing for this population.

## Methods

This systematic review followed Joanna Briggs Institute (JBI) methodology for systematic reviews of effectiveness (Tufanaru et al., 2020) and Preferred Reporting Items for Systematic Reviews (PRISMA) 2020 guidelines (Page et al., 2021). A protocol outlining eligibility criteria, search strategy and methods for data extraction and synthesis was developed prior to conducting the review. The protocol was subsequently registered in the Open Science Framework (OSF), doi: 10.17605/OSF.IO/GRQ75 to enhance transparency.

### Eligibility criteria

#### Population

Studies included adult participants aged 18 years or older, any gender, with a faecal elimination ostomy. Individuals with a prior history of any other type of ostomy were excluded.

#### Intervention

Eligible studies evaluated nurse-led interventions or structured intervention programmes delivered by nurses, with a comparison group and experimental (randomised controlled trials [RCTs], non-randomised trials, quasi-experimental studies, case–control studies or cohort studies)

Interventions included, but were not limited to those described by Bozkul et al. (2024), Burch et al. (2021), Magtoto (2023), Minooee (2021), Ratliff et al. (2021), the Registered Nurses Association of Ontario (2019) and Sivapuram (2022):

- Education on self-care, use of ostomy appliances and management of complications (knowledge provision, instruction and practical training).
- Counselling on the selection and use of ostomy appliances, tailored to the individual’s preferences and clinical needs.
- Hygiene care of the ostomy and peristomal skin.
- Person-centred care, including individualised interventions and emotional support.
- Development of a therapeutic relationship between the nurse and the individual with an ostomy to promote self-care behaviours.

#### Comparation intervention

Studies with passive comparators (no intervention or standard care) or active comparators (alternative or modified interventions) were included.

#### Outcomes

Quantitative studies were included if they reported self-efficacy or psychosocial adjustment, assessed with validated instruments (scales, questionnaires or structured forms) or clinical evaluations with predefined criteria (Tufanaru et al., 2020).

## Search strategy

The search strategy followed the JBI’s three-step approach. Firstly, a preliminary search was conducted in CINAHL Complete and MEDLINE to identify relevant keywords and index terms. Secondly, a comprehensive search was completed on 14 December 2024 in CINAHL Complete (via EBSCOhost), MEDLINE (via EBSCOhost), Psychology and Behavioural Sciences Collection, Scopus, Web of Science and the Cochrane Library, using identified keywords and subject headings (MeSH, CINAHL Headings) combined with Boolean operators. Thirdly, the reference lists of included studies were screened for additional relevant publications. Table 1 presents the full search strategy.

**Table 1.** Search strategy.

P	(‘Ostomy’ OR ‘Surgical Stoma’ OR ‘Colostomy’ OR ‘Ileostomy’ OR ‘Enterostomy’ OR ‘Cecostomy’ OR ‘transversostomy’ OR ‘Bowel ostomy’ OR ‘stoma’)
I	(‘Patient Education’ OR ‘Health Education’ OR ‘Ostomy Care’ OR ‘Nursing Care’ OR ‘Nursing support’ OR ‘Nursing, Practical’ OR ‘nursing intervention*’ OR cognitive OR behave* OR motivational OR psych*)
C	Not defined
O	(‘self management’ OR ‘Self Care’ OR ‘Self Efficacy’ OR Empowerment OR ‘Patient Participation’ OR ‘ostomy adjustment’ OR adjustment OR adaptation OR ‘Patient Autonomy’ OR ‘self-administration’ OR ‘self-monitoring’ OR compliance OR adherence OR ‘self-care behavior*’ OR ‘self-care maintenance’ OR ‘self-care management’ OR ‘illness belief*’ OR ‘illness representation*’ OR ‘attitude to health’ OR ‘health knowledge’)

Note. ^*^indicates truncation used in the database search strategy to retrieve multiple word endings.

The outcomes included in the search strategy reflect core dimensions of self-efficacy and psychosocial adjustment in individuals with ostomy. Terms such as *self-care, self-management, self-efficacy* and *empowerment* represent the ability to manage their condition independently, supported by nurse-led interventions. The Common-Sense Model of Self-Regulation distinguishes behavioural aspects (adherence, self-monitoring and self-care maintenance), from cognitive-emotional influences (illness beliefs, attitudes and health knowledge), which shape motivation and decision-making (Leventhal et al., 2016). Nurse-led interventions foster these outcomes through guidance, emotional support and empowerment (Bird et al., 2023; Chabal et al., 2021; Grant et al., 2013; He et al., 2021; Schott et al., 2022; Schreiber, 2016; Sousa and Santos, 2019).

### Study selection

All references were uploaded to Rayyan, a web-based tool for systematic review screening and reference management, for management and duplicate removal. Titles and abstracts were screened independently by two reviewers, full texts of relevant studies were assessed likewise disagreements were resolved by discussion or a third reviewer.

The selection process was documented in a Preferred Reporting Items for Systematic reviews and Meta-Analyses (PRISMA) 2020 flow diagram, reporting numbers identified, screened, included and excluded, with reasons for exclusion. Table 2 presents the inclusion and exclusion criteria.

**Table 2.** Inclusion and exclusion criteria.

Category	Inclusion criteria	Exclusion criteria
Participants	Adults (⩾18 years old), Individuals with a bowel ostomy	Individuals with prior ostomies of any type
Intervention	Nurse-led interventions or structured intervention programmes delivered by nurses	
Comparison intervention	Standard care, no intervention, or alternative intervention	No comparator
Outcomes	Psychosocial adjustment, Self-efficacy	
Types of studies	Randomised controlled trials, quasi-experimental trials, non-randomised trials, case–control studies	Other study designs, review
Other	English, Spanish or Portuguese, full-text available	Protocol, ongoing study, dissertation, journal paper, conference paper, report, editorial, letter, news, book, promotional or advertising paper

### Data synthesis and analysis

#### Selection process

**Figure 1.**
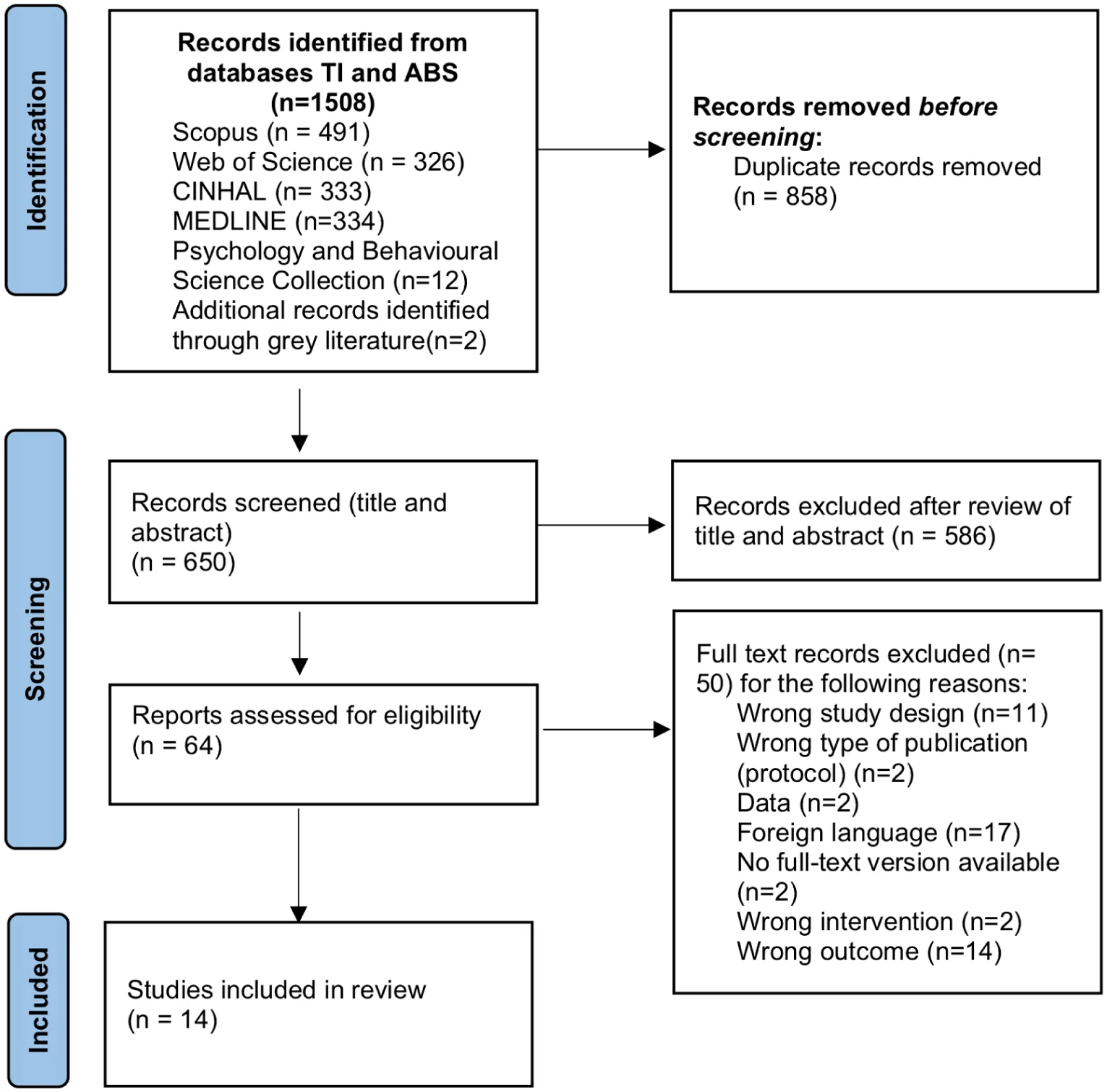
PRISMA flow diagram.

During full-text screening, 64 papers were assessed for eligibility. Of these, 49 papers were excluded for reasons including ineligible study design, outcomes not aligned with the review objectives (not assessing self-efficacy or psychosocial adjustment), language not meeting inclusion criteria and absence of full-text availability. Details of the screening and exclusion process are shown in the PRISMA 2020 flow diagram (Figure 1).

A summary of the included studies and participant characteristics is presented in Table 3.

**Table 3.** Summary of the included studies.

Title	Author (Year)	Country	Methodology	Sample (*n*)	Basic findings
The effect of online training-based continuous nursing care for rectal cancer-patients undergoing permanent colostomy	Huang et al. (2021)	China	Non-randomised controlled trial (RCT)	119	*Assessment points*: Baseline and 6 months. *Self-efficacy*: improved in both groups over 6 months, with significantly greater gains in the intervention group than in the control group.
Pilot trial of a STOMA psychosocial intervention programme for colorectal cancer patients with stomas	Lim et al. (2019)	Singapore	Pilot RCT	53	*Assessment points*: Baseline, mid-intervention measurement (day of discharge), 4 weeks after discharge and 4 months after discharge. *Self-efficacy*: improved across follow-up in both groups, with consistently greater gains in the intervention group, although between-group differences were not statistically significant.
The effects of a nurse-led discharge planning on the health outcomes of colorectal cancer patients with stomas: A randomised controlled trial	Lin et al. (2024)	China	Parallel-arm RCT	160	*Assessment points*: Baseline, immediate post-test, 1 and 3 months. *Self-efficacy*: improved significantly in the intervention group compared with the control group across all follow-up time points up to 3 months.
Effect of using a simulation device for ostomy self-care teaching in Iran: A pilot, randomised clinical trial	Pouresmail et al. (2019)	Iran	Single-blind RCT	46	*Assessment points*: Baseline, 1 week and 45 days after the fourth session. *Self-efficacy*: improved in both groups, with significantly greater gains in the intervention group at post-intervention and follow-up. Psychosocial adjustment also improved over time in both groups, although no significant between-group differences were found.
Effects of the frequency of ostomy management reinforcement education on self-care knowledge, self-efficacy and ability of stoma appliance change among Korean hospitalised ostomates	Seo (2019)	South Korea	Controlled pretest–posttest study	60	*Assessment points*: 7 days. *Self-efficacy*: was significantly higher in both intervention groups compared with the control group at 7 days, with no significant difference between two-session and three-session programmes.
Effects of a home care mobile app on the outcomes of discharged patients with a stoma: a randomised controlled trial	Wang et al. (2018)	China	Single-blind RCT	212	*Assessment points*: Baseline, 1 and 3 months and 6 months. *Self-efficacy*: Significant improvements were observed in the intervention group compared with the control group across all follow-up points up to 6 months. *Psychosocial adjustment*: improved significantly in the intervention group compared with the control group at 1, 3 and 6 months.
Effects of the frequency of ostomy management reinforcement education on self-care knowledge, self-efficacy and ability of stoma appliance change among Korean hospitalised ostomates	Xu and Zhou (2023)	China	Retrospective comparative study	187	*Assessment points*: Before and after intervention. *Self-efficacy*: The experimental groups 1 and 2 showed greater improvement in self-efficacy and ability to change stoma appliances. Two sessions were sufficient to achieve these outcomes. Control group show improvement but significantly less than in the experimental groups
Effect of Self-efficacy Intervention on Quality of Life of Patients With Intestinal Stoma	Xu et al. (2018)	China	RCT	48	*Assessment points*: 10 days, 1 month and 3 months. *Self-efficacy*: No significant between-group differences were observed after 10 days; however, the intervention group showed significantly greater improvements in self-efficacy than the control group at 1 and 3 months.
The effects of patient care results of applied nursing intervention to individuals with stoma according to the health belief model	Cengiz et al. (2020)	Turkey	Quasi-experimental controlled study	61	*Assessment points*: Baseline and 6 months *Psychosocial adjustment*: IG: Significant improvement (*p* < 0.00), median 36.00; mean 44.00 CG: No significant changes. *Acceptance*: mean rank IG 25.38-> 28.75; CG 23.63-> 21.98 *Anxiety*: mean rank IG 16.00-> 10.00; CG 13.00-> 7.00 *Social adjustment*: mean rank IG 13.50 -> 10.00; CG 12.00->8.00 *Anger*: mean rank IG 5.50->4.00; CG 4.00->4.00
Health related quality of life may increase when patients with a stoma attend patient education – a case–control study	Danielson and Rosenberg (2014)	Denmark	Case–control study	50	*Assessment points*: Baseline, 3 and 6 months. *Psychosocial adjustment*: improved significantly in the intervention group over 6 months, whereas no significant changes were observed in the control group. Improvements were also reported in acceptance, anxiety, social adjustment and anger-related outcomes.
Effect of a stoma nursing care programme on the adjustment of patients with an ostomy	Fernandes and Brito (2020)	Portugal	Quasi-experimental controlled study	105	*Assessment points*: Baseline, 1 and 6 months. *Psychosocial adjustment*: No significant between-group differences were observed at 1-month post-discharge; however, the intervention group showed significantly better psychosocial adjustment at 6 months. Improvements were particularly evident in self-concept, positive acceptance and self-care domains
The effect of phone counselling service on adaptation to stoma and quality of life among patients with intestinal stoma: a randomised controlled trial	Taylan and Aksoy (2021)	Turkey	RCT	60	*Assessment points*: Baseline, 6 and 10 weeks. *Psychosocial adjustment*: improved significantly in the intervention group from baseline to 10 weeks, with progressive gains observed at 6 and 10 weeks.
Effect of a mobile patient education application on adjustment to stoma and development of peristomal skin lesions: a quasi-experimental study	Yiğitoğlu and Sendir (2021)	Turkey	Quasi-experimental study	60	*Assessment points*: Baseline, 1 and 3 months. *Psychosocial adjustment*: improved significantly in the intervention group at 1 and 3 months, whereas no significant changes were observed in the control group. Significant gains were also reported in social engagement at 3 months
Post-discharge health education for patients with enterostomy: a nationwide interventional study	Zhou et al. (2023)	China	Quasi-experimental study	4201	*Assessment points*: Baseline and 23 weeks. *Psychosocial adjustment*: improved significantly from discharge to 23 weeks after the intervention. Significant improvements were also observed in emotional well-being and social life adaptation

#### Data extraction process

Data extraction was performed by two reviewers using a standardised form that was piloted on a subset of included studies (n = 2) to ensure clarity and consistency. Extracted data included study characteristics (authorship, year, country, period, design, objectives), participants (sample, control/comparator, intervention, eligibility), intervention details (content, duration, timing, instrument), outcomes, findings and limitations. Discrepancies were resolved by discussion and rechecking original papers when needed.

#### Synthesis methods

Given high heterogeneity (χ^2^ = 31.32, df = 4, *p* < 0.00001; *I*^2^ = 87%), no meta-analysis was performed. Instead, a narrative synthesis followed Bazeley’s (2020) approach of systematic, comparative and integrative analysis: (i) extracting relevant results considering methodological and clinical contexts; (ii) comparing findings to identify convergences/divergences and (iii) integrating results into themes of psychosocial adjustment and self-efficacy. Direction of effect was determined based on between-group inferential statistics (*p*-values or confidence intervals) reported in the primary studies. Statistically significant improvements in intervention groups relative to controls were classified as positive effects.

To enhance coherence, three frameworks were applied: theoretical domains framework (TDF) (Atkins et al., 2017; Cane et al., 2012) to categorise behavioural domains (knowledge, skills, beliefs, social influences); the World Health Organization (WHO, 2023) framework to classify delivery (face-to-face, digital/remote, hybrid) and the transitional care model (TCM) (Naylor et al., 2018) to categorise delivery stage (preoperative, discharge, follow-up).

Additionally, nurse-led interventions were categorised by TCM (Naylor et al., 2018): preoperative preparation, transitional care at discharge/follow-up and sustained support. This classification provided a structured view of how interventions are sequenced and tailored across care, clarifying when they are most effective in supporting adaptation among people with a stoma.

This combined approach ensured a coherent, theory-informed synthesis while preserving study diversity. It offered a structured lens to examine sequencing and tailoring of interventions, clarifying when nurse-led care best supports adjustment and self-efficacy in people with a stoma.

#### Reporting bias assessment

To assess risk of bias, all studies were appraised for methodological quality by two reviewers using JBI critical appraisal checklists (Tufanaru et al., 2020), selected by study design.

The results were compared between reviewers, and any disagreements were resolved through discussion. Agreement between reviewers (*k* = 0.93) indicated an *almost perfect* level of agreement (Landis and Koch, 1977).

Methodological quality was assessed to support interpretation of the findings rather than as a criterion for study exclusion. All eligible studies were retained for data extraction and synthesis. Detailed appraisal results are presented in Supplemental Material 1. Analysing by question, quality assessment showed lowest scores (Supplemental Material 2). Some questions scored 0%, but most scored 100%, supporting inclusion of all papers.

Overall, the majority of studies were judged to have a moderate risk of bias, mainly related to lack of blinding, limited strategies to address confounding and some uncertainty in allocation procedures, whereas other methodological domains were generally well addressed.

## Results

### Overview of included studies

Fourteen studies (2014–2024) were included, with 5680 participants with an intestinal elimination ostomy. Reported mean ages ranged from 45.8 to 67 years, and the median age across studies was 58.1 years (IQR 50.2–61.0). Age data were not reported in one study. Most studies were conducted in China (*n* = 6) and Turkey (*n* = 3), with others in Denmark (*n* = 1), Portugal (*n* = 1), Iran (*n* = 1), Singapore (*n* = 1) and South Korea (*n* = 1). The most frequent designs were RCTs (*n* = 6) and quasi-experimental studies (*n* = 6), followed by case–control (*n* = 2). No study compared outcomes between emergency and elective ostomy creation.

Nurse-led interventions included structured education with follow-up, mobile health apps for post-discharge care, simulation training and scheduled consultations. Duration ranged from 3 weeks to 6 months, with follow-up from perioperative to 1 year post-surgery. The most common outcome measures were the Stoma Self-Efficacy Scale (SSES) (*n* = 7) and the Ostomy Adjustment Inventory-23 (OAI-23) (*n* = 6). Other measures are listed in Supplemental Material 3.

Due to the high methodological and clinical heterogeneity among the included studies, a formal certainty assessment of the body of evidence (e.g. the Grading of Recommendations Assessment, Development and Evaluation [GRADE] approach) was not undertaken. Instead, the level of confidence in the review findings was based on the methodological rigour of the included studies, as assessed by the JBI critical appraisal tools and on the coherence of results across studies.

Analysis with Review Manager (RevMan) revealed very high methodological and clinical heterogeneity: Chi^2^ = 31.32 (df = 4, *p* < 0.00001) and *I*^2^ = 87% for self-efficacy, plus substantial heterogeneity for psychosocial adjustment (only four studies provided sufficient data). This heterogeneity made a valid meta-analysis infeasible due to lack of homogeneity required for robust synthesis. Forest plots illustrating between-study variability are therefore provided in the Supplemental Materials 4 and 5.

Consequently, a narrative synthesis was undertaken to integrate study findings and describe the direction of effect of nurse-led interventions on self-efficacy and psychosocial adjustment. This approach preserved the richness of diverse study designs while respecting methodological and clinical variability.

#### Effect of nurse-led intervention on self-efficacy behaviours and psychosocial adjustment

Across the included studies, nurse-led interventions were predominantly multicomponent and designed to support both self-care behaviours and psychosocial adjustment during the postoperative period and transition to home. As summarised in Table 4, interventions combined educational, psychosocial, cognitive or cognitive-behavioural, functional training and symptom management strategies.

**Table 4.** Summarises the methodological characteristics and intervention contents.

Study	Educational interventions	Psychosocial interventions	Cognitive interventions	Functional and training interventions	Symptom management interventions
Cengiz et al. (2020)	X	X	X	X	X
Danielsen and Rosenberg (2014)	X	X	X	X	
Fernandes and Brito (2020)	X	X	X	X	X
Huang et al. (2021)	X	X	X	X	X
Lim et al. (2019)	X	X	X	X	X
Lin et al. (2024)	X	X	X	X	X
Pouresmail et al. (2019)	X	X	X	X	X
Seo (2019)	X		X	X	X
Taylan and Aksoy (2021)	X	X	X	X	X
Wang et al. (2018)	X	X	X	X	X
Xu and Zhou (2023)	X	X	X	X	X
Xu et al. (2018)	X	X	X	X	X
Yiğitoğlu and Sendir (2021)	X	X	X	X	X
Zhou et al. (2023)	X	X	X	X	X

Across studies, interventions were multicomponent, usually combining education, behavioural strategies and follow-up. All studies included an educational intervention, although format and intensity varied. Six studies (Danielsen and Rosenberg, 2014; Fernandes and Brito, 2020; Huang et al., 2021; Lim et al., 2019; Lin et al., 2024) described structured programmes integrating multiple approaches to strengthen self-efficacy and psychosocial adjustment. This category was not exclusive, as several also incorporated functional training, digital tools or psychosocial components.

Follow-up support was a common feature. Five studies (Cengiz et al., 2020; Danielsen and Rosenberg, 2014; Lim et al., 2019; Taylan and Aksoy, 2021; Xu et al., 2018; Zhou et al., 2023) used both telephone and face-to-face, whereas one study (Fernandes and Brito, 2020) relied only on in-person. Five studies (Pouresmail et al., 2019; Seo, 2019; Wang et al., 2018; Xu and Zhou, 2023; Yiğitoğlu and Şendir, 2021) integrated digital platforms or mobile applications, whereas two studies (Lin et al., 2024; Huang et al., 2021) combined digital with telephone and in-person follow-up. Discharge planning was reported in one study (Lin et al., 2024) and simulation training in another (Pouresmail et al., 2019). All compared interventions with routine care, with Xu and Zhou (2023) and Seo (2019) testing two groups.

Nurse-led interventions mainly activated knowledge, skills, decision-making/attention and behavioural regulation, with consistent focus on self-efficacy. However, intentions/goals, reinforcement, emotion and social/professional role and identity were underused TDF domains, showing opportunities to strengthen psychosocial adjustment and sustain self-care behaviours. Table 5 shows the TDF domains identified.

**Table 5.** Map of interventions to TDF domain (Atkins et al., 2017; Cane et al., 2012).

Categories of nurse-led interventions	Educational		Functional and training		Cognitive or cognitive–behavioural			Symptom management				Psychosocial		
TDF Authors (year)	Knowledge	Memory, attention and decision processes	Behavioural regulation	Skills	Beliefs about Capabilities	Optimism	Beliefs about Consequences	Intentions	Goals	Reinforcement	Environmental context and resources	Social influences	Emotion	Social/professional role and identity
Cengiz et al. (2020)	X	X	X	X			X					X	X	
Danielsen and Rosenberg (2014)	X	X	X	X	X						X	X	X	
Fernandes and Brito (2020)	X	X	X	X	X						X	X		
Huang et al. (2021)	X	X	X	X	X						X	X	X	
Lim et al. (2019)	X	X	X	X	X						X	X	X	
Lin et al. (2024)	X	X	X	X	X						X	X		
Pouresmail et al. (2019)	X	X	X	X	X						X	X		
Seo (2019)	X	X	X	X	X						X	X		
Taylan and Aksoy (2021)	X		X		X						X	X		
Wang et al. (2018)	X	X	X	X	X						X	X	X	
Xu and Zhou (2023)	X	X		X	X						X	X	X	
Xu et al. (2018)	X	X	X	X	X						X	X	X	
Yiğitoğlu and Sendir (2021)	X	X	X	X	X						X	X		
Zhou et al. (2023)	X	X	X	X	X						X	X	X	

Table 6 synthesises the effects of nurse-led interventions on self-efficacy and psychosocial adjustment by type, delivery mode, timing and professional involvement.

**Table 6.** Synthesis of the effects of nurse-led interventions on self-efficacy and psychosocial adjustment.

Nurse-led interventions	Self-efficacy		Adjustment	
	No effect	Favourable effect	No effect	Favourable effect
Type				
Educational	Lim et al. (2019)	Huang et al. (2021); Lin et al. (2024); Pouresmail et al. (2019); Seo (2019); Wang et al. (2018); Xu and Zhou (2023); Xu et al. (2018)	Danielsen and Rosenberg (2014); Pouresmail et al. (2019)	Cengiz et al. (2020); Fernandes and Brito (2020); Taylan and Aksoy (2021); Wang et al. (2018); Yiğitoğlu and Sendir (2021); Zhou et al. (2023)
Functional training	Lim et al. (2019)	Huang et al. (2021); Lin et al. (2024); Pouresmail et al. (2019); Seo (2019); Wang et al. (2018); Xu and Zhou (2023); Xu et al. (2018)	Danielsen and Rosenberg (2014); Pouresmail et al. (2019)	Cengiz et al. (2020); Fernandes and Brito (2020); Taylan and Aksoy (2021); Wang et al. (2018); Yiğitoğlu and Sendir (2021); Zhou et al. (2023)
Cognitive or cognitive-behavioural	Lim et al. (2019)	Huang et al. (2021); Lin et al. (2024); Pouresmail et al. (2019); Seo (2019); Wang et al. (2018); Xu and Zhou (2023); Xu et al. (2018)	Danielsen and Rosenberg (2014); Pouresmail et al. (2019)	Cengiz et al. (2020); Fernandes and Brito (2020); Taylan and Aksoy (2021); Wang et al. (2018); Yiğitoğlu and Sendir (2021); Zhou et al. (2023).
Symptom management	Lim et al. (2019)	Huang et al. (2021); Lin et al. (2024); Pouresmail et al. (2019); Seo (2019); Wang et al. (2018); Xu and Zhou (2023); Xu et al. (2018)	Danielsen and Rosenberg (2014); Pouresmail et al. (2019)	Fernandes and Brito (2020); Taylan and Aksoy (2021); Wang et al. (2018); Yiğitoğlu and Sendir (2021); Zhou et al. (2023)
Psychosocial	Lim et al. (2019)	Huang et al. (2021); Lin et al. (2024); Pouresmail et al. (2019); Seo (2019); Wang et al. (2018); Xu and Zhou (2023); Xu et al. (2018)	Danielsen and Rosenberg (2014); Pouresmail et al. (2019)	Fernandes and Brito (2020); Cengiz et al. (2020); Taylan and Aksoy (2021); Wang et al. (2018); Yiğitoğlu and Sendir (2021); Zhou et al. (2023)
Mode of delivery				
Individual face-to-face encounters		Pouresmail et al. (2019); Seo (2019)	Pouresmail et al. (2019)	Fernandes and Brito (2020)
Digital/remote interventions		Wang et al. (2018)	Danielsen and Rosenberg (2014)	Cengiz et al. (2020); Taylan and Aksoy (2021); Wang et al. (2018); Yiğitoğlu and Sendir (2021)
Hybrid models	Lim et al. (2019)	Huang et al. (2021); Lin et al. (2024); Xu and Zhou (2023); Xu et al. (2018)		Zhou et al. (2023)
Timing				
Preoperative preparation and Engagement	Lim et al. (2019)	Lin et al. (2024)		Fernandes and Brito (2020)
Transitional care at hospital discharge	Lim et al. (2019)	Lin et al. (2024); Pouresmail et al. (2019); Seo (2019); Xu and Zhou (2023)	Pouresmail et al. (2019)	Fernandes and Brito (2020)
Follow-up and sustained support	Lim et al. (2019)	Huang et al. (2021); Lin et al. (2024); Wang et al. (2018); Xu et al. (2018)	Danielsen and Rosenberg (2014)	Cengiz et al. (2020); Fernandes and Brito (2020); Taylan and Aksoy (2021); Wang et al. (2018); Yiğitoğlu and Sendir (2021); Zhou et al. (2023)
Professionals involved				
Unidisciplinary	Lim et al. (2019)	Pouresmail et al. (2019); Seo (2019); Wang et al. (2018); Xu et al. (2018)	Pouresmail et al. (2019)	Cengiz et al. (2020); Fernandes and Brito (2020); Taylan and Aksoy (2021); Wang et al. (2018); Yiğitoğlu and Sendir (2021); Zhou et al. (2023)
Multidisciplinary		Huang et al. (2021); Lin et al. (2024); Xu and Zhou (2023)	Danielsen and Rosenberg (2014)	

Educational, training, cognitive-behavioural, symptom management and psychosocial approaches generally showed positive outcomes, though some studies (Lim et al., 2019) reported non-significant effects, suggesting that context and design influence results.

Hybrid delivery (face-to-face, telephone, digital) produced consistent benefits, whereas single-mode formats were more variable. Follow-up and sustained support yielded the strongest effects, underscoring continuity beyond discharge. Preoperative and transitional interventions also showed benefits, though less robust.

Most interventions were unidisciplinary, nurse-led and effective for both outcomes. Multidisciplinary models offered broader psychosocial benefits, reflecting the value of team-based care. Overall, nurse-led interventions were most effective when multicomponent, hybrid in delivery and sustained over time.

Across studies, favourable effects were more consistent for self-efficacy than for psychosocial adjustment. Improvements in self-efficacy were most consistently reported when interventions combined education and skills training with structured follow-up, particularly in hybrid delivery formats (face-to-face plus telephone/digital support). By contrast, effects on psychosocial adjustment were more variable, with more consistent outcomes when interventions included explicit psychosocial components, were delivered over a longer follow-up period or involved multidisciplinary input. Interventions limited to preoperative or transitional phases showed benefits, but these were less robust than programmes that extended into post-discharge follow-up and sustained support.

##### Effects on self-efficacy

**Figure 2.**
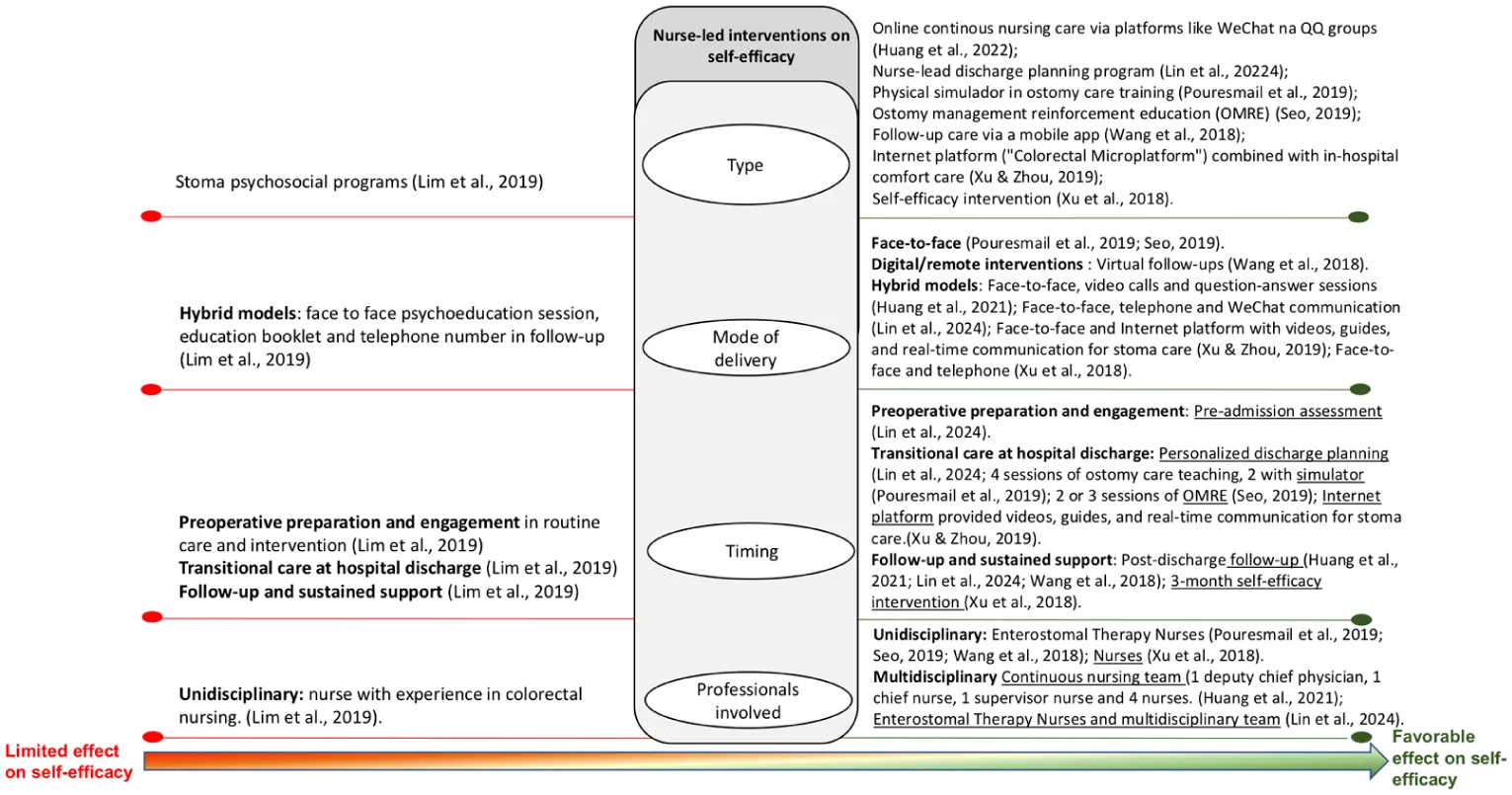
Effects of nurse-led interventions on self-efficacy.

Overall, nurse-led interventions showed a consistent beneficial effect on self-efficacy behaviours. Detailed findings are presented in Supplemental Material 6, whereas Figure 2 provides a synthesis of the effects of nurse-led interventions on self-efficacy in individuals with a bowel ostomy, organised by intervention type, delivery mode, timing and professionals involved. Improvements were most evident in interventions combining education and functional training with structured follow-up (Huang et al., 2021; Lin et al., 2024; Wang et al., 2018; Xu et al., 2018; Xu and Zhou, 2023).

Simulator-based training and practical education produced short-term improvements in confidence and self-management skills (Pouresmail et al., 2019; Seo, 2019). Digital interventions, including mobile applications and remote monitoring, demonstrated sustained improvements in self-efficacy over time (Wang et al., 2018). Hybrid models combining face-to-face and remote support showed the most consistent and durable effects (Huang et al., 2021; Lin et al., 2024; Xu et al., 2018; Xu and Zhou, 2023). In contrast, an intervention focused primarily on psychosocial components without strong educational or technical elements did not significantly improve self-efficacy (Lim et al., 2019).

Interventions at hospital discharge with follow-up were particularly effective for long-term self-efficacy (Huang et al., 2021; Wang et al., 2018; Lin et al., 2024). Preoperative interventions helped prepare patients and families (Lin et al., 2024; Lim et al., 2019), though effects were less consistent. In Wang et al. (2018), preoperative preparation was routine care, whereas in Xu et al. (2018) routine care were not specified.

Most interventions were unidisciplinary, reinforcing nurses’ central role in self-efficacy support (Pouresmail et al., 2019; Seo, 2019; Wang et al., 2018; Xu et al., 2018). Multidisciplinary teams with physicians, psychologists and nutritionists produced broader, integrated effects, highlighting the value of interprofessional collaboration (Huang et al., 2021; Lin et al., 2024).

##### Effects on psychosocial adjustment

**Figure 3.**
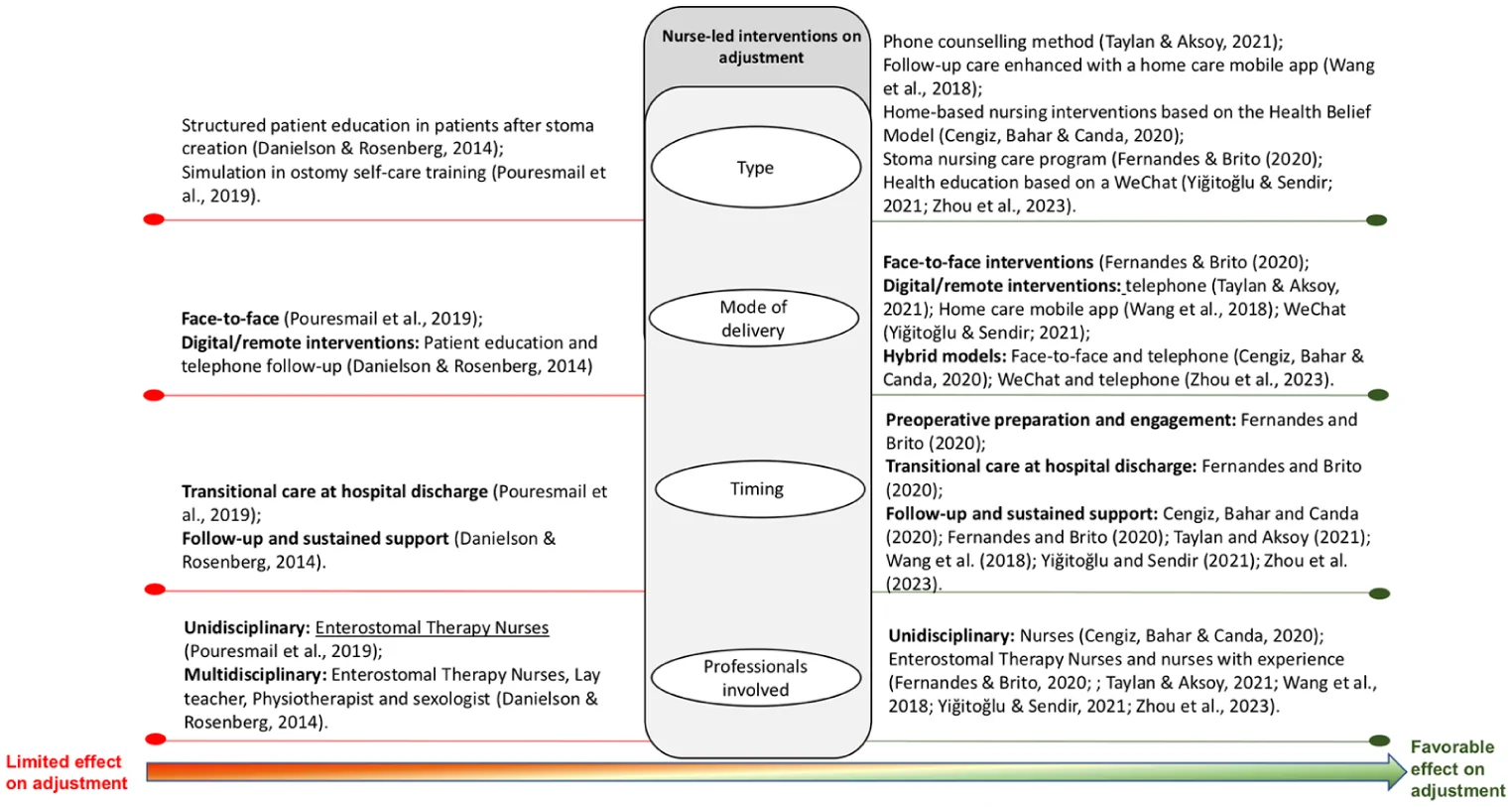
Effect of nurse-led interventions on psychosocial adjustment.

Nurse-led interventions also demonstrated a beneficial, though more variable, effect on psychosocial adjustment (Supplemental Material 7). Positive outcomes were more consistently reported in interventions that included explicit psychosocial components and sustained follow-up. Figure 3 illustrates the effects of nurse-led interventions on psychosocial adjustment.

Structured stomatherapy programmes initiated preoperatively and continued into follow-up significantly improved psychosocial adjustment and self-care (Fernandes and Brito, 2020). Digital interventions, including WeChat-based programmes and mobile applications, improved psychosocial adjustment, self-efficacy and self-care (Wang et al., 2018; Yiğitoğlu and Şendir, 2021; Zhou et al., 2023). Telephone counselling showed short-term improvements in psychosocial well-being and quality of life (Taylan and Aksoy, 2021).

By contrast, simulator-based interventions improved technical confidence but did not produce sustained psychosocial adjustment (Pouresmail et al., 2019). Educational interventions involving professionals and lay teachers showed some benefits, although group differences were less evident at follow-up (Danielsen and Rosenberg, 2014).

Overall, findings show nurse-led interventions positively impact self-efficacy and psychosocial adjustment in people with a stoma. Across studies, educational, functional training, cognitive-behavioural and psychosocial strategies proved most effective when delivered as multicomponent programmes, often reinforced through hybrid delivery models and sustained follow-up support. Improvements in self-efficacy typically emerged earlier and with greater consistency, providing patients with the technical and behavioural competencies required for independent stoma management. Psychosocial adjustment, though more variable, improved most with comprehensive, sustained interventions, underscoring continuity of care. The interplay between these outcomes underscores that self-efficacy and psychosocial adjustment are mutually reinforcing. Greater confidence in self-care supports adaptation, whereas emotional acceptance and social reintegration provide the motivational foundation required to sustain long-term self-management. Collectively, the evidence underscores the central role of nurses in delivering holistic, person-centred care that supports both the practical and emotional transitions of living with a stoma.

#### Influence of surgical conditions associated with ostomy formation

The influence of the surgical condition on intervention effects on self-efficacy behaviours and psychosocial adjustment was not explicitly examined in the included studies. Although participants underwent bowel ostomy formation in different clinical contexts, including colorectal cancer and other gastrointestinal conditions, studies did not report outcomes according to surgical indication or provide subgroup analyses (e.g. elective vs emergency surgery) (Huang et al., 2021; Lin et al., 2024; Xu et al., 2018; Xu and Zhou, 2023).

Reporting of clinical characteristics was heterogeneous across studies, and no stratified analyses were identified for self-efficacy or psychosocial adjustment outcomes according to surgical condition (Danielsen and Rosenberg, 2014; Lim et al., 2018; Wang et al., 2018). Therefore, no conclusions can be drawn regarding whether the effects of nurse-led interventions on self-efficacy behaviours and psychosocial adjustment differ according to the surgical condition associated with bowel ostomy formation.

#### Influence of delivered mode of interventions

The mode of delivery appeared to influence the effects of nurse-led interventions on self-efficacy behaviours and psychosocial adjustment. *Individual face-to-face interventions* included inpatient education, functional training or outpatient follow-up, emphasising direct patient–nurse interaction for skills, support and personalised education (Fernandes and Brito, 2020; Pouresmail et al., 2019; Seo, 2019).

*Digital or remote interventions* relied primarily on mobile applications, online platforms or telephone counselling (Danielsen and Rosenberg, 2014; Taylan and Aksoy, 2021; Wang et al., 2018; Yiğitoğlu and Sendir, 2021). These approaches expanded nurse-led care beyond hospitals, enabling continuous monitoring and support at home and in the community.

*Hybrid interventions* were most common (*n* = 6), combining face-to-face, telephone and/or digital components to maximise continuity and flexibility of delivery (Cengiz et al., 2020; Zhou et al., 2023; Huang et al., 2021; Lim et al., 2018; Lin et al., 2024; Xu et al., 2018; Xu and Zhou, 2023). By integrating formats, hybrid models addressed the complex, evolving needs of people with a stoma during transition from hospital to home.

#### Influence of timing of interventions

Timing influenced intervention outcomes. *Preoperative interventions* were less frequent and mainly supported preparation, engagement and expectation management (Fernandes and Brito, 2020; Lim et al., 2018; Lin et al., 2024) to prepare patients and families, foster engagement and adjust expectations before surgery.

*Transitional care interventions*, focusing on discharge planning and coordination between hospital and community, were more common. They aimed to ensure safe discharge and continuity of support (Pouresmail et al., 2019; Seo, 2019; Xu and Zhou, 2023).

Most studies emphasised *follow-up support* after discharge, either through face-to-face visits, remote monitoring or hybrid approaches (Cengiz et al., 2020; Huang et al., 2021; Wang et al., 2018; Yiğitoğlu and Sendir, 2021; Zhou et al., 2023). Such interventions focused on reinforcing self-care, identifying complications early and sustaining psychosocial adjustment.

#### Influence of professionals involved in the interventions

Nurse-led interventions were delivered either *unidisciplinarily*, with nurses solely responsible, or in *multidisciplinary* formats involving physicians, psychologists, dietitians or peer support. This distinction reflects both nursing autonomy and the degree of integration and teamwork underpinning care.

Most studies used *unidisciplinary* approaches (Cengiz et al., 2020; Fernandes and Brito, 2020; Pouresmail et al., 2019; Seo, 2019; Taylan and Aksoy, 2021; Wang et al., 2018; Xu et al., 2018; Yiğitoğlu and Sendir, 2021; Zhou et al., 2023 ), highlighting the central role of nurses in providing technical and psychosocial care through education, training and follow-up.

Fewer studies reported *multidisciplinary interventions* (Danielsen and Rosenberg, 2014; Huang et al., 2021; Lin et al., 2024; Xu and Zhou, 2023), where nurses collaborated with other professionals to address broader dimensions of care, such as psychological counselling, medical management or nutritional support. These collaborative approaches reflect efforts to integrate stoma care into a holistic, team-based healthcare model.

## Discussion

### Nurse-led interventions and their effects on self-efficacy and psychosocial adjustment

The findings of this review indicate that nurse-led interventions can effectively support both self-efficacy behaviours and psychosocial adjustment in people living with a bowel ostomy. Across studies, multimodal interventions combining educational, behavioural, psychosocial and functional training strategies demonstrated the most consistent benefits. These findings are consistent with those of Kittscha et al. (2022), who suggested that adjustment to life with an ostomy is facilitated by increased knowledge, independence in stoma management and continued access to specialised ostomy care nurses. Similarly, Bozkul et al. (2024) confirmed the positive impact of nurse-led interventions on self-efficacy, while emphasising the ongoing need for psychosocial support.

The stronger and more consistent improvements observed in self-efficacy may reflect the nature of the interventions delivered. Most interventions targeted knowledge acquisition, technical skills, behavioural regulation and confidence in self-management, domains closely aligned with Bandura’s concept of self-efficacy and with the TDF. Evidence from other chronic conditions also reinforces the impact of nurse-led interventions on self-efficacy; in patients with acute coronary syndrome, a randomised controlled trial showed that a nurse-led counselling and education programme, delivered through two face-to-face sessions and two telephone-based sessions using a person-centred care approach, improved cardiac self-efficacy and supported recovery compared with usual care (Bagheri et al., 2022).

In contrast, psychosocial adjustment appeared more variable and required longer-term interventions addressing emotional adaptation, body image, social reintegration and identity reconstruction. This finding is consistent with Goodman et al. (2022), who observed that self-efficacy tends to improve earlier than psychosocial adaptation, and with Vonk-Klaassen et al. (2016), who highlighted the persistent influence of complications, altered body image and social restrictions on long-term quality of life.

The findings further suggest that self-efficacy and psychosocial adjustment are interdependent processes. Greater confidence in ostomy self-care may facilitate adaptation and autonomy, whereas emotional acceptance and social reintegration provide the motivational basis required to sustain self-management behaviours over time. Kittscha et al. (2022) similarly argued that psychosocial adjustment involves a multifaceted and ongoing process of rebuilding self-image and integrating the ostomy into everyday life.

From a behavioural perspective, the interventions primarily activated TDF domains related to knowledge, skills, memory and decision processes, behavioural regulation and beliefs about capabilities. These domains appear central to promoting self-care competence and self-efficacy in ostomy management. However, domains related to emotion, reinforcement, intentions/goals and social/professional role and identity were comparatively underexplored. This finding suggests that current nurse-led interventions may prioritise technical and educational dimensions of care over emotional adaptation and identity reconstruction.

Future interventions could therefore benefit from incorporating behaviour change techniques (BCTs) more explicitly linked to emotional coping, goal-setting, feedback, peer role-modelling and social support. Nadal et al. (2024) identified goals and planning, feedback and monitoring, knowledge enhancement and social support as the most effective BCT clusters for sustaining health behaviour change. Integrating these techniques into ostomy care programmes may strengthen psychosocial adjustment and support more durable self-management outcomes.

The findings also reinforce the value of theoretical frameworks in intervention design. Previous studies applying the TDF and behaviour change wheel (BCW) demonstrated that theory-informed interventions can improve implementation and behaviour change outcomes (Richardson et al., 2019; Smith et al., 2019). More recently, Nourse et al. (2024) showed that combining BCW with user-centred design principles can enhance the development of digital interventions by aligning behavioural determinants with patient needs and preferences. This integrated approach appears particularly promising for ostomy self-care interventions.

### Influence of surgical conditions associated with ostomy formation

The second research question could only be partially answered, as none of the included studies explicitly examined whether the effects of nurse-led interventions differed according to the surgical condition associated with ostomy formation. Although participants underwent ostomy surgery in diverse clinical contexts, including colorectal cancer and other gastrointestinal conditions, studies did not report subgroup analyses comparing elective and emergency surgery or different surgical indications.

This represents an important gap in the evidence, particularly given that emergency ostomy formation is frequently associated with reduced preoperative preparation, increased psychological distress, greater uncertainty and more abrupt transitions to self-care. Individuals undergoing emergency surgery may therefore experience different psychosocial and educational needs compared with those receiving planned elective procedures. The absence of stratified analyses limits understanding of how intervention effectiveness may vary across surgical contexts and restricts the development of tailored nurse-led approaches.

Future research should prioritise comparative studies examining emergency versus elective ostomy formation and evaluate whether intervention timing, intensity or psychosocial support requirements differ according to surgical trajectory and clinical context.

### Influence of mode of delivery

The findings related to the third research question suggest that the mode of intervention delivery influences both self-efficacy and psychosocial adjustment outcomes. Hybrid interventions combining face-to-face contact with telephone or digital follow-up demonstrated the most consistent and sustained benefits across studies. These approaches appear particularly valuable in supporting continuity of care during the transition from hospital to home.

Digital interventions, including mobile applications, online platforms and telehealth support, expanded nurse-led care beyond the hospital setting and enabled ongoing monitoring, education and psychosocial support within community settings. Similar findings have been reported in broader chronic disease management literature. For example, Kılıç and Karadağ (2020) demonstrated stronger effects in diabetes self-management when digital tools were combined with in-person care. In ostomy care, Krouse et al. (2024) identified subgroup and dose–response benefits associated with telehealth interventions, whereas Iovino et al. (2024) suggested that telehealth education may provide continuity comparable to traditional face-to-face approaches. Kittcha et al. (2022) indicate that follow-up beyond face-to-face visits using phone-based technology improves adjustment to an ostomy because they offer frequent access to an ostomy nurse.

However, the review also suggests that digital interventions alone may not fully address the emotional and relational dimensions of adaptation. Free et al. (2013) reported only modest benefits associated with mobile health interventions, emphasising the continued importance of direct professional contact. Furthermore, digital and hybrid approaches may be influenced by barriers related to digital literacy, older age, socio-economic inequalities and access to internet or technological resources (Odendaal et al., 2020). These factors should be considered when implementing mHealth-supported ostomy care programmes.

### Influence of timing of interventions

The timing of interventions also appeared to influence outcomes, thereby partially answering the fourth research question. Most studies focused on postoperative and follow-up phases, whereas preoperative interventions were comparatively uncommon. When implemented, preoperative interventions primarily aimed to prepare patients and families, foster engagement, normalise expectations and reduce uncertainty before surgery.

The concept of prehabilitation in ostomy care remains relatively underdeveloped despite evidence suggesting that early psychological and educational preparation may facilitate recovery and adaptation (Chabal et al., 2021; Registered Nurses’ Association of Ontario, 2019; Sivapuram, 2022). Clark et al. (2023) similarly noted that structured psychological preparation before surgery remains largely absent, despite evidence of benefits for anxiety reduction and quality of life. Organisational barriers may partly explain this gap, as Matsubara and Hirohata (2024) reported limited availability of preoperative outpatient education among WOC (Wound, Ostomy and Continence) nurses in Japan.

Findings confirm that self-efficacy gains typically appear earlier, whereas psychosocial adjustment requires longer and more complex interventions. This suggests that, unlike self-efficacy, which may respond to shorter and skill-focused interventions, psychosocial adjustment is more likely to require intensive, multicomponent and sustained strategies that address emotional, social and identity-related processes over time. Goodman et al. (2022) also observed consistent self-efficacy improvements but variable effects on adjustment. Vonk-Klaassen et al. (2016) highlighted how complications, body image concerns and social restrictions persistently reduce quality of life, explaining the challenge of sustaining psychosocial gains. Kittscha et al. (2022) suggested that the reestablishment of body and self-image is linked to acceptance and influences psychosocial adjustment, this constitutes a multifaceted and ongoing process.

Future research should therefore prioritise the development and evaluation of structured nurse-led preoperative and transitional care programmes integrating education, psychosocial preparation, skills rehearsal and long-term follow-up.

### Influence of professionals involved in the interventions

The fifth research question highlighted differences between unidisciplinary and multidisciplinary approaches. Most interventions were delivered in unidisciplinary nurse-led formats, reinforcing the central role of nurses in providing education, technical training, behavioural support and psychosocial care throughout the adaptation process.

These findings highlight nursing autonomy and the importance of specialised stomatherapy nursing in promoting self-management and continuity of care. However, multidisciplinary approaches involving physicians, psychologists, nutritionists and peer support appeared to offer broader psychosocial benefits by addressing emotional, social and identity-related dimensions more comprehensively.

Danielsen and Rosenberg (2014), for example, demonstrated the value of combining professional and lay support within structured educational interventions. Such collaborative models align with person-centred and holistic approaches to chronic care, recognising that adjustment to an ostomy extends beyond technical self-care and encompasses emotional adaptation, social participation and identity reconstruction.

### Strengths and limitations

This review has several strengths. To our knowledge, it is the first to systematically examine nurse-led interventions on both self-efficacy and psychosocial adjustment in people with a stoma. Analysing these outcomes together offers a comprehensive view of how educational, behavioural and psychosocial strategies support adaptation. The review also followed a rigorous methodology with structured searches, clear eligibility criteria and systematic data extraction, ensuring transparency and reproducibility. Frameworks such as the WHO digital health model and TCM provided conceptual structure and highlighted delivery mechanisms.

Limitations include heterogeneity in study design, sample size, follow-up and outcomes, reducing comparability and generalisability. Publication bias cannot be ruled out and most studies were single-country, limiting cultural applicability. Intervention diversity also prevented meta-analysis, restricting conclusions to narrative synthesis. In addition, language bias may be present, as studies published in languages other than English, Spanish or Portuguese were excluded, which may have resulted in omission of relevant evidence.

### Implications

The findings of this review highlight the importance of preparing nurses to deliver multicomponent interventions that integrate technical, educational, behavioural and psychosocial dimensions of ostomy care. Nursing education programmes should strengthen competencies related to self-management support, communication, psychosocial assessment, behavioural change strategies and digital health delivery. Greater emphasis should also be placed on preparing nurses to support emotional adaptation, body image concerns, identity reconstruction and long-term adjustment following ostomy formation. Educational curricula and continuing professional development programmes may benefit from incorporating evidence-informed frameworks such as the TDF, BCTs and person-centred approaches to support the design and implementation of holistic nurse-led interventions.

The findings support the integration of nurse-led, multicomponent interventions into routine ostomy care pathways. Interventions combining education, skills training, psychosocial support, behavioural strategies and structured follow-up demonstrated the most consistent benefits for self-efficacy and psychosocial adjustment. Psychosocial support should therefore be integrated alongside technical training rather than treated as a secondary component of care.

The review further suggests that hybrid delivery models combining face-to-face, telephone and digital support may strengthen continuity of care and sustain long-term adaptation. Seamless collaboration between hospital and community nurses appears particularly important in supporting transitions from hospital to home and maintaining continuity across preoperative, transitional and follow-up phases. Incorporating digital and hybrid models may additionally improve accessibility, flexibility and continuity of support for patients and families across settings.

From a behavioural perspective, clinical programmes may benefit from explicitly integrating TDF-informed strategies and monitoring behavioural mediators such as intentions/goals, self-monitoring, emotional regulation and identity or peer connection alongside traditional clinical outcomes. Using brief logic models and standardised intervention descriptions may also help clarify active intervention components and improve implementation consistency across settings.

The findings underscore the need for healthcare systems and policy-makers to support continuity of ostomy care across preoperative, postoperative, discharge and follow-up phases. Policies should facilitate the integration of nurse-led and hybrid follow-up interventions into standard ostomy care pathways. At the same time, healthcare systems should ensure equitable access to digital and remote support while maintaining non-digital alternatives for individuals with limited digital literacy, restricted technological access or socio-economic vulnerabilities. Organisational investment in specialised stomatherapy nursing services and multidisciplinary collaboration may further strengthen person-centred and transitional care delivery.

Future research should prioritise the development and evaluation of theoretically informed interventions that clearly specify intervention components, delivery mode, timing, intensity and hypothesised mechanisms of action. Standardised reporting and the use of logic models may improve comparability across studies and strengthen evidence synthesis. In addition to clinical outcomes, future trials should monitor behavioural mediators aligned with underexplored TDF domains, including intentions and goals, emotional regulation, reinforcement, self-monitoring and identity reconstruction. Comparative studies examining emergency versus elective ostomy formation are also needed to better understand how surgical context influences intervention needs and effectiveness. Further research should additionally explore the long-term sustainability, accessibility and equity implications of digital and hybrid models of care.

## Conclusion

This review shows that nurse-led interventions are effective drivers of behaviour change in people with a bowel ostomy. Across studies, programmes activated core TDF mechanisms – knowledge, skills, decision processes and behavioural regulation -producing early, consistent gains in self-efficacy. Psychosocial adjustment improved less consistently and appears to require additional strategies such as goal setting, reinforcement, emotional support and work on identity.

Findings support routine implementation of multicomponent, TDF-informed programmes starting early (ideally preoperatively or before discharge), embedding action planning and providing structured follow-up with feedback. Hybrid models enhanced accessibility and durability, whereas multidisciplinary inputs offered added value in addressing emotional and social challenges.

Self-efficacy gains typically emerged earlier than psychosocial improvements, which demand longer-term strategies. Face-to-face training remained central for skills and therapeutic alliance, whereas digital tools extended continuity, especially in hybrid designs. Interventions starting during hospitalisation or at discharge and continuing into follow-up yielded the most stable effects; evidence for preoperative programmes is still limited.

Overall, nurses play a pivotal role in promoting both self-efficacy and psychosocial adjustment. Future studies should use larger, multicentre designs, examine dosage and timing, include TDF-based mediators, extend follow-up to 6–12 months and assess cost-effectiveness and digital inclusion. Strengthening these elements will help translate early self-efficacy gains into lasting psychosocial adjustment and sustained self-care.

**Key points for policy, practice and research**

Policy

- Policies should support the integration of nurse-led, multicomponent interventions into standard stoma care pathways, beginning in the preoperative phase and ensuring continuity across discharge and follow-up.
- Health systems should ensure access to hybrid follow-up options (face-to-face combined with telephone/digital support) while providing non-digital alternatives to minimise inequities in access.

Practice

- Nurse-led, multicomponent interventions combining education, skills training and follow-up show the most consistent benefits for self-efficacy and psychosocial adjustment.
- Psychosocial support should be integrated alongside technical training and not treated as secondary.
- Hybrid delivery models, led by nurses and delivered to patients and families across settings, were associated with stronger and more sustained outcomes than single-mode approaches.

Research

- Future studies should use standardised reporting and brief logic models to specify active ingredients (components), delivery mode, timing, dose/intensity and hypothesised mechanisms of action.
- In addition to outcomes, future trials should monitor behavioural mediators aligned with underused TDF domains (e.g. intentions/goals, self-monitoring, emotion regulation and identity/peer connection) to clarify how interventions achieve sustained effects.

## Supplemental Material

sj-docx-1-jrn-10.1177_17449871261462845 – Supplemental material for The effect of nurse-led interventions in self-efficacy and psychosocial adjustment of individuals with bowel ostomy: a systematic review Supplemental material, sj-docx-1-jrn-10.1177_17449871261462845 for The effect of nurse-led interventions in self-efficacy and psychosocial adjustment of individuals with bowel ostomy: a systematic review by Joana Portela, Graça Melo, Tânia Mendes and Helga Rafael Henriques in Journal of Research in Nursing

sj-docx-2-jrn-10.1177_17449871261462845 – Supplemental material for The effect of nurse-led interventions in self-efficacy and psychosocial adjustment of individuals with bowel ostomy: a systematic review Supplemental material, sj-docx-2-jrn-10.1177_17449871261462845 for The effect of nurse-led interventions in self-efficacy and psychosocial adjustment of individuals with bowel ostomy: a systematic review by Joana Portela, Graça Melo, Tânia Mendes and Helga Rafael Henriques in Journal of Research in Nursing

sj-docx-3-jrn-10.1177_17449871261462845 – Supplemental material for The effect of nurse-led interventions in self-efficacy and psychosocial adjustment of individuals with bowel ostomy: a systematic review Supplemental material, sj-docx-3-jrn-10.1177_17449871261462845 for The effect of nurse-led interventions in self-efficacy and psychosocial adjustment of individuals with bowel ostomy: a systematic review by Joana Portela, Graça Melo, Tânia Mendes and Helga Rafael Henriques in Journal of Research in Nursing

sj-docx-6-jrn-10.1177_17449871261462845 – Supplemental material for The effect of nurse-led interventions in self-efficacy and psychosocial adjustment of individuals with bowel ostomy: a systematic review Supplemental material, sj-docx-6-jrn-10.1177_17449871261462845 for The effect of nurse-led interventions in self-efficacy and psychosocial adjustment of individuals with bowel ostomy: a systematic review by Joana Portela, Graça Melo, Tânia Mendes and Helga Rafael Henriques in Journal of Research in Nursing

sj-docx-7-jrn-10.1177_17449871261462845 – Supplemental material for The effect of nurse-led interventions in self-efficacy and psychosocial adjustment of individuals with bowel ostomy: a systematic review Supplemental material, sj-docx-7-jrn-10.1177_17449871261462845 for The effect of nurse-led interventions in self-efficacy and psychosocial adjustment of individuals with bowel ostomy: a systematic review by Joana Portela, Graça Melo, Tânia Mendes and Helga Rafael Henriques in Journal of Research in Nursing

sj-jpg-4-jrn-10.1177_17449871261462845 – Supplemental material for The effect of nurse-led interventions in self-efficacy and psychosocial adjustment of individuals with bowel ostomy: a systematic review Supplemental material, sj-jpg-4-jrn-10.1177_17449871261462845 for The effect of nurse-led interventions in self-efficacy and psychosocial adjustment of individuals with bowel ostomy: a systematic review by Joana Portela, Graça Melo, Tânia Mendes and Helga Rafael Henriques in Journal of Research in Nursing

sj-jpg-5-jrn-10.1177_17449871261462845 – Supplemental material for The effect of nurse-led interventions in self-efficacy and psychosocial adjustment of individuals with bowel ostomy: a systematic review Supplemental material, sj-jpg-5-jrn-10.1177_17449871261462845 for The effect of nurse-led interventions in self-efficacy and psychosocial adjustment of individuals with bowel ostomy: a systematic review by Joana Portela, Graça Melo, Tânia Mendes and Helga Rafael Henriques in Journal of Research in Nursing
