# Supplementary material for: The effect of nurse-led interventions in self-efficacy and psychosocial adjustment of individuals with bowel ostomy: a systematic review
Source: J Res Nurs. 2026 Aug 20:17449871261462845. Online ahead of print. doi: 10.1177/17449871261462845 (PMC13498689; doi:10.1177/17449871261462845)
Supplement: Supplementary file 1 [file sj-docx-2-jrn-10.1177_17449871261462845.docx]

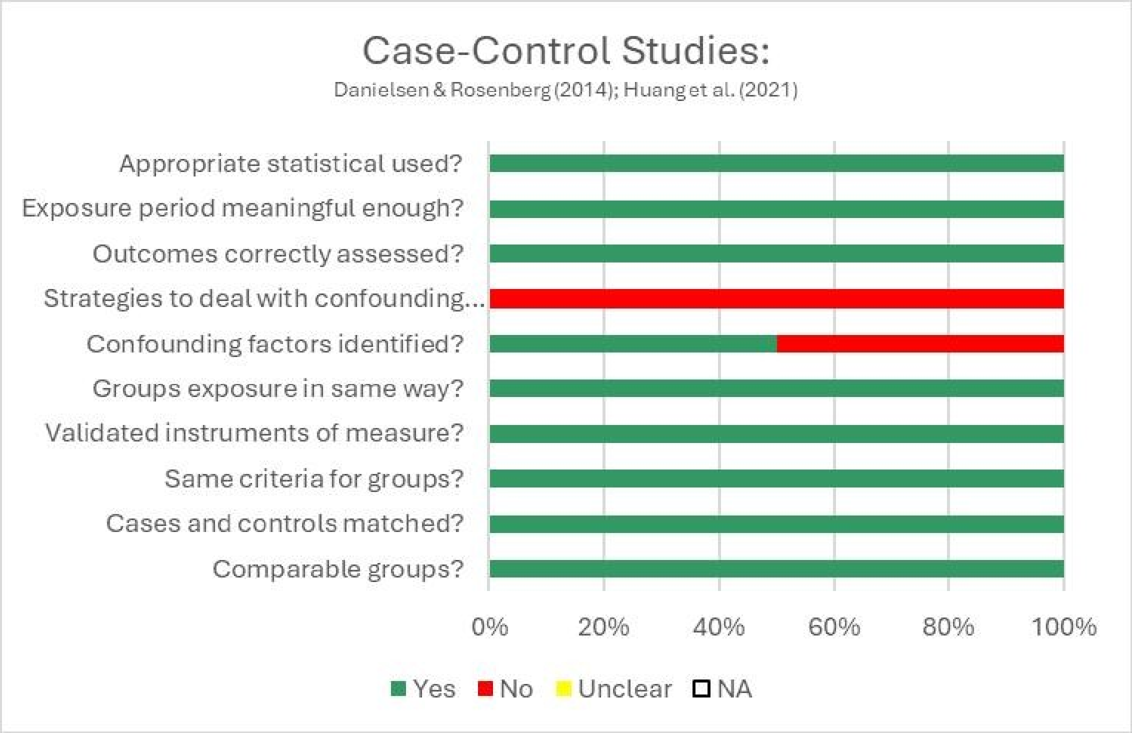
Case-Control Studies:
Danielsen & Rosenberg (2014); Huang et al. (2021)
Appropriate statistical used?
Exposure period meaningful enough?
Outcomes correctly assessed?
Strategies to deal with confounding...
Confounding factors identified?
Groups exposure in same way?
Validated instruments of measure?
Same criteria for groups?
Cases and controls matched?
Comparable groups?
0%
20%
40%
60%
80%
100%
Yes
No
Unclear
NA

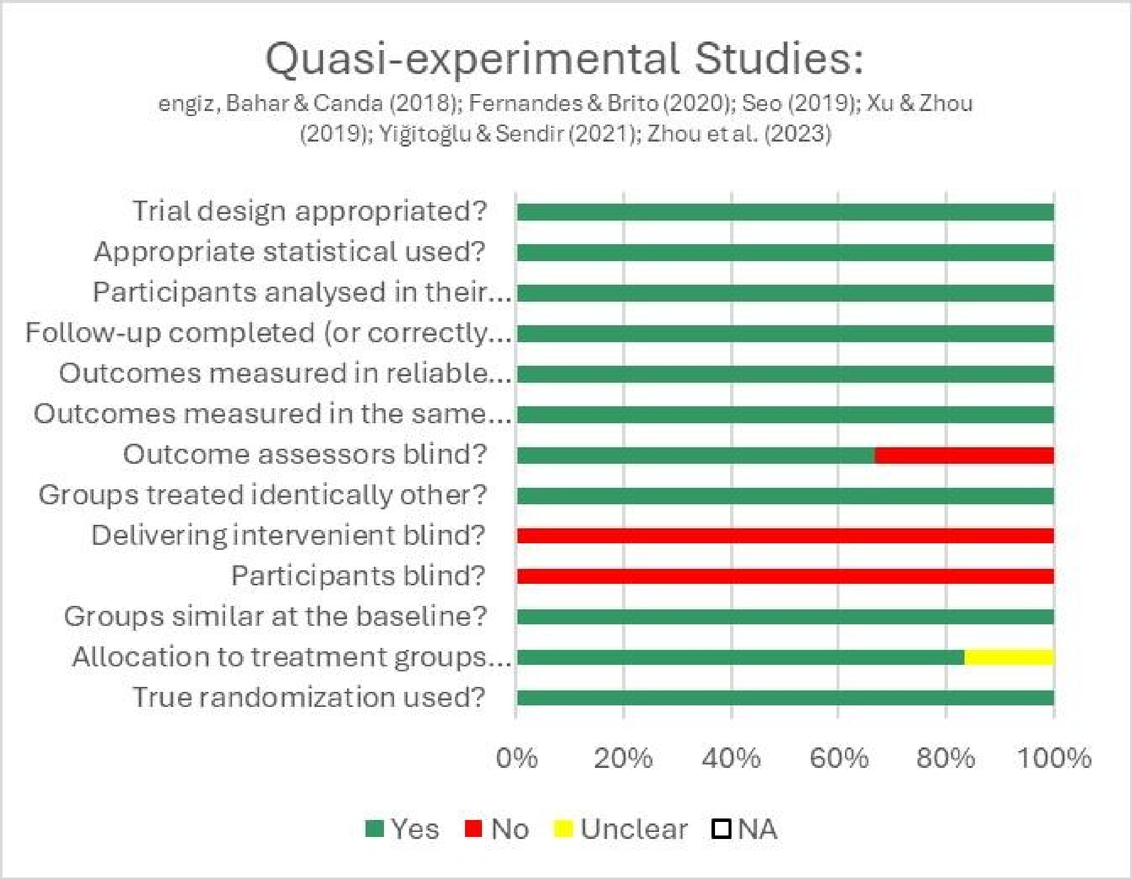
Quasi-experimental Studies:
engiz, Bahar & Canda (2018); Fernandes & Brito (2020); Seo (2019); Xu & Zhou (2019); Yiğitoğlu & Sendir (2021); Zhou et al. (2023)
Trial design appropriated?
Appropriate statistical used?
Participants analysed in their...
Follow-up completed (or correctly...
Outcomes measured in reliable...
Outcomes measured in the same...
Outcome assessors blind?
Groups treated identically other?
Delivering intervenient blind?
Participants blind?
Groups similar at the baseline?
Allocation to treatment groups...
True randomization used?
0%
20%
40%
60%
80%
100%
Yes
No
Unclear
NA

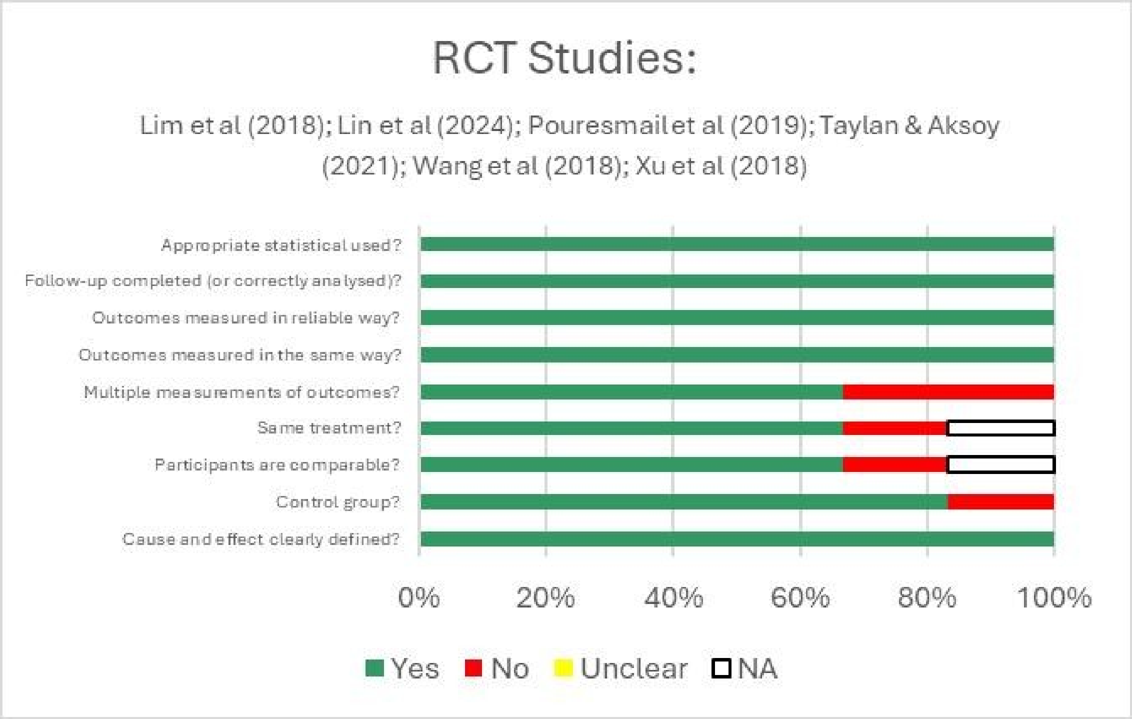

RCT Studies:
Lim et al (2018); Lin et al (2024); Pouresmail et al (2019); Taylan & Aksoy (2021); Wang et al (2018); Xu et al (2018)
Appropriate statistical used?
Follow-up completed (or correctly analysed)?
Outcomes measured in reliable way?
Outcomes measured in the same way?
Multiple measurements of outcomes?
Same treatment?
Participants are comparable?
Control group?
Cause and effect clearly defined?
0%
20%
40%
60%
80%
100%
Yes
No
Unclear
NA
