# Supplementary material for: The effect of nurse-led interventions in self-efficacy and psychosocial adjustment of individuals with bowel ostomy: a systematic review
Source: J Res Nurs. 2026 Aug 20:17449871261462845. Online ahead of print. doi: 10.1177/17449871261462845 (PMC13498689; doi:10.1177/17449871261462845)
Supplement: Supplementary file 1 [file sj-docx-1-jrn-10.1177_17449871261462845.docx]

Supplemental material 2- Quality Assesment Summary

|  | Question:  ID: | Comparable groups? | Cases and controls matched? | Same criteria for groups? | Validated instruments of measure? | Groups exposure in same way? | Confounding factors identified? | Strategies to deal with confounding factors? | Outcomes correctly assessed? | Exposure period meaningful enough? | Appropriate statistical used? |  | | | Quality Level | | |  |
| --- | --- | --- | --- | --- | --- | --- | --- | --- | --- | --- | --- | --- | --- | --- | --- | --- | --- | --- |
|  |  |  |  |  |  |  |  |  |  |  |  |  |  |  | Reviewer A | Reviewer B | Consensus |  |
| Case Control | Danielsen & Rosenberg (2014) | Y | Y | Y | Y | Y | Y | N | Y | Y | Y |  |  |  | 90% | 100% | 90% |  |
|  | Huang et al. (2021) | Y | Y | Y | Y | Y | N | N | Y | Y | Y |  |  |  | 80% | 80% | 80% |  |
|  | | Cause and effect clearly defined? | Control group? | Participants are comparable? | Same treatment (except intervention)? | Multiple measurements of outcomes? | Outcomes measured in the same way? | Outcomes measured in reliable way? | Follow-up completed (or correctly analysed)? | Appropriate statistical used? |  |  |  |  |  |  |  |  |
| Quasi-Experimental | Cengiz, Bahar & Canda (2018) | Y | Y | Y | N | Y | Y | Y | Y | Y |  |  |  |  | 89% | 89% | 89% |  |
|  | Fernandes & Brito (2020) | Y | Y | N | Y | N | Y | Y | Y | Y |  |  |  |  | 100% | 78% | 78% |  |
|  | Seo (2019) | Y | Y | Y | Y | Y | Y | Y | Y | Y |  |  |  |  | 100% | 100% | 100% |  |
|  | Xu & Zhou (2019) | Y | Y | Y | Y | Y | Y | Y | Y | Y |  |  |  |  | 100% | 100% | 100% |  |
|  | Yiğitoğlu & Sendir (2021) | Y | Y | Y | Y | N | Y | Y | Y | Y |  |  |  |  | 89% | 100% | 89% |  |
|  | Zhou et al. (2023) | Y | N | NA | NA | Y | Y | Y | Y | Y |  |  |  |  | 75% | 86% | 86% |  |
|  | | True randomization used? | Allocation to treatment groups concealed? | Groups similar at the baseline? | Participants blind? | Delivering intervenient blind? | Groups treated identically other? | Outcome assessors blind? | Outcomes measured in the same way? | Outcomes measured in reliable way? | Follow-up completed (or correctly analysed)? | Participants analysed in their groups? | Appropriate statistical used? | Trial design appropriated? |  |  |  |  |
| RCT  ied Clinical Trial | Lim et al. (2018) | Y | Y | Y | N | N | Y | Y | Y | Y | Y | Y | Y | Y | 85% | 85% | 85% |  |
|  | Lin et al. (2024) | Y | Y | Y | N | N | Y | Y | Y | Y | Y | Y | Y | Y | 85% | 85% | 85% |  |
|  | Pouresmail et al. (2019) | Y | Y | Y | N | N | Y | Y | Y | Y | Y | Y | Y | Y | 85% | 85% | 85% |  |
|  | Taylan & Aksoy (2021) | Y | U | Y | N | N | Y | Y | Y | Y | Y | Y | Y | Y | 77% | 77% | 77% |  |
|  | Wang et al. (2018) | Y | Y | Y | N | N | Y | N | Y | Y | Y | Y | Y | Y | 77% | 77% | 77% |  |
|  | Xu et al. (2018) | Y | Y | Y | N | N | Y | N | Y | Y | Y | Y | Y | Y | 77% | 77% | 77% |  |
