# Supplementary material for: The effect of nurse-led interventions in self-efficacy and psychosocial adjustment of individuals with bowel ostomy: a systematic review
Source: J Res Nurs. 2026 Aug 20:17449871261462845. Online ahead of print. doi: 10.1177/17449871261462845 (PMC13498689; doi:10.1177/17449871261462845)
Supplement: Supplementary file 3 [file sj-docx-3-jrn-10.1177_17449871261462845.docx]

Table - Outcomes measure used

|  | Outcomes measure used | n | |
| --- | --- | --- | --- |
| **Selfcare** | Ostomy Self-care ability questionnaire (Zhou et al., 2023) | 1 | 10 |
|  | Stoma Self-Efficacy Scale (Lim et al., 2018; Lin et al., 2024; Pouresmail et al., 2019; Seo, 2018; Wang et al., 2018; Xu & Zhou, 2023; Xu et al., 2018) | 7 |  |
|  | General Self-Efficacy Scale (GSES) (Huang et al., 2021) | 1 |  |
|  | Selfcare Ability Scale (ESCA) (Huang et al., 2021) | 1 |  |
| **Ostomy Adjusment** | EOAS (Fernandes & Brito, 2020) | 1 | 8 |
|  | Ostomy Adjustment Inventory-20 (Zhou et al., 2023) | 1 |  |
|  | Ostomy Adjustment Inventory-23 (Cengiz, Bahar & Canda, 2018; Pouresmail et al., 2019; Taylan & Aksoy, 2021; Wang et al., 2018; Yiğitoğlu & Sendir, 2021) | 5 |  |
|  | OAS (Danielsen & Rosenberg, 2014) | 1 |  |
| **Anxiety and depression** | Anxiety and depression Hospital Anxiety and Depression Scale (HADS) (Lim et al., 2018) | 1 | 4 |
|  | Selfcare-rating Anxiety Scale (SAS) e Self-rating Depression Scale (SDS) (Huang et al., 2021) | 1 |  |
|  | Hamilton Anxiety Scale (HAMA) and Hamilton Depression Scale (HAMD); (Zhou et al., 2023; Xu & Zhou, 2023) | 2 |  |
| **Health care survey** | Short Form Health Care Survey (SF-36) (Huang et al., 2021; Danielsen & Rosenberg, 2014) | 2 | 2 |
| **Quality of life** | Stoma Quality of Life Scale (Lin et al., 2024; Cengiz, Bahar & Canda, 2018) | 2 | 6 |
|  | QoL (Lim et al., 2018) | 1 |  |
|  | QLE-C30 (Xu et al., 2018) | 1 |  |
|  | COH-QoL-OQ (Xu & Zhou, 2023) | 1 |  |
|  | Adaptation of Quality Life Scale (Taylan & Aksoy, 2021) | 1 |  |
| **Outros** | Quality Discharge teaching scale (Lin et al., 2024) | 1 |  |
|  | Readiness for Hosital Discharge scale (Lin et al., 2024) | 1 |  |
|  | Peristomal Skin Lesions Assessment and classification instrument (Yiğitoğlu & Sendir, 2021) | 1 |  |
|  | Ostomy Complications Severity Index (Cengiz, Bahar & Canda, 2018) | 1 |  |
|  | Acceptance of stoma (Lim et al., 2018) | 1 |  |
|  | Selfcare knowledge level (Seo, 2018) | 1 |  |
|  | Measurement tool for ability change stoma appliance (Seo, 2018) | 1 |  |
|  | Brief fatigue inventory (Xu & Zhou, 2023) | 1 |  |
