# Supplementary material for: The effect of nurse-led interventions in self-efficacy and psychosocial adjustment of individuals with bowel ostomy: a systematic review
Source: J Res Nurs. 2026 Aug 20:17449871261462845. Online ahead of print. doi: 10.1177/17449871261462845 (PMC13498689; doi:10.1177/17449871261462845)
Supplement: Supplementary file 4 [file sj-docx-6-jrn-10.1177_17449871261462845.docx]

Table - Main findings on self-efficacy

| **Authors (Year)** | **Instrument used** | **Assessment points** | **Main findings on self-efficacy** |
| --- | --- | --- | --- |
| Huang et al. (2021) | GSES | Baseline and 6 months | Observation group: Significant improvement (18.97 ± 1.64 → 23.18 ± 2.03, p < 0.05).  Control group: Modest improvement (19.14 ± 1.77 → 21.06 ± 1.91, p < 0.05). |
| Lim et al. (2018) | SSES | Baseline, Mid-intervention measurement (day of discharge), 4 weeks after discharge and 4 months after discharge. | Higher improvement in intervention group across all time points. No significant differences between groups over time. |
| Lin et al. (2024) | SSES | Baseline, immediate post-test, 1 month and 3 months | Stoma Self-Efficacy: Higher improvement in intervention group across all time points (p<0.001). |
| Pouresmail et al. (2019) | SSES | Baseline, 1 week and 45  days after the fourth session. | Self-efficacy improved from baseline to follow-up in both groups (P <.0001). Excluding age, the SSES showed a significant difference in the intervention group from baseline (69.30 ± 12.07), fourth session (103.82 ± 15.81), to follow-up (111.43 ± 15.79) (F = 3.183; P = .008).  Significant difference between groups in terms of the self-efficacy score fourth session (P = .004) and follow-up (P <.0001). |
| Seo (2019) | SSES | 7 days | Significantly higher in experimental groups 1 and 2 compared to the control group (p = 0.001).  No significant difference between experimental group 1 (two sessions) and experimental group 2 (three sessions). |
| Wang al. (2018) | SSES | Baseline, 1 month, 3 months and 6 months | Significant improvement in the intervention group at all follow-up points (p < 0.001).  Moderate improvement in control group.  IG (n = 100) Within group F (p) 682.21 (0.000a) Between groups F (p) 23.16 (0.000a) Interaction effect F (p) 49.58 (0.000a)  Baseline Mean 54,64 SD 15,57  1 month Mean 66,08 SD 12,53  3 month Mean 80,53 SD 9,78  6 month Mean 70.80 SD 7,78  CG: (n = 103) Within group F (p) 682.21 (0.000a) Between groups F (p) 23.16 (0.000a) Interaction effect F (p) 49.58 (0.000a)  Baseline Mean 54,15 SD 19,13  1 month Mean 60,21 SD 16,94  3 month Mean 69,07 SD 14,27  6 month Mean 75,50 SD 13,38 |
| Xu & Zhou (2023) | SSES | Before and after intervention | The experimental groups 1 and 2 showed greater improvement in self-efficacy and ability to change stoma appliances. Two sessions were suficiente to achive these outcomes.  Control group show improvement but significantly less than in the experimental groups |
| Xu el al. (2019) | SSES | 10 days, 1 month and 3 months | After 10 days of intervention no significant difference on the improvement of the self  Efficacy.  Between the two groups after 1 and 3 months was statistically significant (p< 0.05).  Improvement in 3 months after the nursing intervention (significant statistical difference compared with the control group)  No significant difference among the scores of the three time points in the control group. |
