# Supplementary material for: The effect of nurse-led interventions in self-efficacy and psychosocial adjustment of individuals with bowel ostomy: a systematic review
Source: J Res Nurs. 2026 Aug 20:17449871261462845. Online ahead of print. doi: 10.1177/17449871261462845 (PMC13498689; doi:10.1177/17449871261462845)
Supplement: Supplementary file 5 [file sj-docx-7-jrn-10.1177_17449871261462845.docx]

Table - Main findings on psychosocial adjustment

| **Authors (Year)** | **Instrument used** | **Assessment points** | **Main findings on psycosocial adjustment** |
| --- | --- | --- | --- |
| Cengiz, Bahr & Canda (2018) | OAI-23 | Baseline and 6 months | IG: Significant improvement (p < .00), median 36.00-> 44.00  CG: No significant changes.  Acceptance: mean rank IG 25.38-> 28.75; CG 23.63-> 21.98  Anxiety: mean rank IG 16.00-> 10.00; CG 13.00-> 7.00  Social adjustment: mean rank IG 13.50 -> 10.00; CG 12.00->8.00  Anger: mean rank IG 5.50->4.00; CG 4.00->4.00 |
| Danielsen & Rosenberg (2014) | OAS | Baseline, 3 month and 6 month | IG: Significant improvement from baseline to 3 and 6 months (p < 0.001).  CG: No significant changes (p = 0.14).  Baseline scores were lower in the intervention group, but differences were not significant at 6 months. |
| Fernandes & Brito (2020) | EOAS | Baseline, 1 month and 6 months | 1Month Post-Discharge: No significant differences between IG and CG in overall EOAS scores.  Sixth Month Post-Discharge: IG had significantly better overall EOAS scores (p < .001). Improvements observed in self-concept, positive acceptance, and self-care domains (p < .01). CG presente minimal improvements.  Overall  IG: 1month 57.50 (11.05); 6 month 67.07 (11.22)  CG: 1month 58.47 (12.12); 6 month 57.38 (10.42)  Self-concept  IG: 1month 45.15 (20.81); 6 month 53.37 (13.47)  CG: 1month 45.84 (21.00); 6 month 43.17 (13.63)  Positive Acceptance  IG: 1month 56.89 (16.88); 6 month 64.37 (14.75)  CG: 1month 54.29 (12.54); 6 month 54.94 (11.92)  Negative Acceptance  IG: 1month 60.38 (18.64); 6 month 71.61 (15.50)  CG: 1month 65.50 (16.18); 6 month 61.92 (16.95)  Social/religious support  IG: 1month 70.40 (16.88); 6 month 67.66 (13.92)  CG: 1month 80.06 (11.56); 6 month 76.25 (14.40)  Social interaction  IG: 1month 54.01 (14.95); 6 month 36.56 (13.54)  CG: 1month 54.60 (14.92); 6 month 38.11 (12.30)  Self-care  IG: 1month 52.24 (16.65); 6 month 71.23 (19.99)  CG: 1month 50.00 (12.65); 6 month 53.26 (22.95)  Positive Acceptance  IG: 1month 56.89 (16.88); 6 month 64.37 (14.75)  CG: 1month 54.29 (12.54); 6 month 54.94 (11.92) |
| Pouresmail et al. (2019) | OAI-23 | Baseline, 1 week and 45  days after the fourth session | IG: Improved from baseline to follow-up (45 days) (p=0.000). Baseline: Mean 51.04 SD 13.37. 1 week: Mean 60.56 SD 16.41. 45 days: Mean 64.60 SD 16.74  CG: Baseline: Mean 50.82 SD 13.84. 1 week: Mean 53.95 SD 13.22. 45 days: Mean 58.17 SD 13.07.  Improved from baseline to follow-up (p=0.000)  Higher score in IG but no significant difference was found between the 2 groups in terms of mean adjustment scores during the intervention from baseline to follow-up (P = .517) . No significant difference between groups in terms of adjustment score after the 1 week and 45 days |
| Taylan & Aksoy (2021) | OAI-23 | Baseline, 6 week and 10 week | IG: Significant improvement in the test group from baseline to week 10 (p < 0.001). Baseline: Mean 43.67 SD 10.52 Median 40.5 Z (-0,555) 1p 0.579. 6 week: Mean 52.57 SD 7.71 Median 55 Z (-5.859) 1p 0.001; 10 week: Mean 59.30 SD 5.88 Median 58.5 Z (-6.447) 1p 0.001  Acceptance  Baseline: Mean 6.67 SD 3.71 Median 16 Z (-0,883) 1p 0,377; 6 week: Mean 24.60 SD3.47 Median 25 Z (-5.848) 1p 0.001;10 week: Mean 27.47 SD 2.91 Median 27 Z (-6.188) 1p 0.001  Anxious preoccupation  Baseline: Mean 9.70 SD 3.50 Median 9 Z (-0,082) 1p 0,934; 6 week: Mean 12.00 SD 3.03 Median 12,5 Z (-5.237) 1p 0.001; 10ª week: Mean 11.47 SD 2.62 Median 11 Z (-4.306) 1p 0.001  Social engagement  Baseline: Mean 7.27 SD 2.86 Median 7 Z (-0,351) 1p 0,726; 6 week: Mean 6.77 SD 1.81 Median 7 Z (-5.517) 1p; 0.001; 10 week: Mean 8.37 SD 1.59 Median 8.5 Z (-5.736) 1p 0.001  Anger  Baseline: Mean 3.93 SD 1.76 Median 4 Z (-0,442) 1p 0,658; 6 week: Mean 3.70 SD 1.09 Median 4 Z (-3.595) 1p 0.001; 10 week: Mean 4.67 SD 1.47 Median 4 Z (-4.118) 1p 0.001  CG: slower improvement. Baseline: Mean 41.5 SD 8.34 Median 40 Z (-0,555) 1p 0.579. 6 week: Mean 34.10 SD 9.05 Median 33 Z (-5.859) 1p 0.001. 10 week: Mean 43.50 SD 4.70 Median 42 Z (-6.447) 1p 0.001  Acceptance  Baseline: Mean 15.43 SD 4.26 Median 15.5 Z (-0,883) 1p 0,377. 6 week: Mean 16.73 SD 3.87 Median 16.5 Z (-5.848) 1p 0.001. 10 week: Mean 20.63 SD 2.72 Median 20 Z (-6.188) 1p 0.001  Anxious preoccupation  Baseline: Mean 9.60 SD 3.08 Median 9.5 Z (-0,082) 1p 0,934. 6 week: Mean 6.63 SD 2.94 Median 6 Z (-5.237) 1p 0.001. 10 week: Mean 8.37 SD 2.24 Median 8 Z (-4.306) 1p 0.001  Social engagement  Baseline: Mean 6.93SD 2.53 Median 6.5 Z (-0,351) 1p 0,726. 6 week: Mean 3.87 SD 1.28 Median 4 Z (-5.517) 1p 0.001. 10 week: Mean 5.00 SD 1.23 Median 5 Z (-5.736) 1p 0.001  Anger  Baseline: Mean 3.70 SD 1.70 Median 4 Z (-0,442) 1p 0,658  6 week: Mean 2.57 SD 1.07 Median 2.5 Z (-3.595) 1p 0.001  10 week: Mean 3.27 SD 0.58 Median 3 Z (-4.118) 1p 0.001 |
| Wang et al. (2018) | OAI-23 | Baseline, 1 month, 3 months and 6 months | Intervention group showed significant improvement over control at 1 month (p < 0.001), 3 months (p < 0.001), and 6 months (p < 0.001).  IG: (n = 100) Within group F (p) 1112.74 (0.000a) Between groups F (p) 81.21 (0.000a) Interaction effect F (p) 79.56 (0.000a)  Baseline Mean 39.31 SD 10.47  1 month Mean 51.32 SD 8.43  3 month Mean 64.36 SD 5.94  6 month Mean 70.80 SD 4.64  CG: (n = 103) Within group F (p) 1112.74 (0.000a) Between groups F (p) 81.21 (0.000a) Interaction effect F (p) 79.56 (0.000a)  Baseline Mean 36,34 SD 12,07  1 month Mean 41,23 SD 11.43  3 month Mean 49,81 SD 10,41  6 month Mean 54,54 SD 10,48 |
| Yiğitoğlu & Sendir (2021) | OAI-23 | Baseline, 1 month and 3 monts | IG: 52.5 (range, 41–67) and 56 (range, 35–77) in the first and third months, respectively. Tstatistically significant (P= .05). In the third month in the social engagement subscale scores compared with the initial evaluation (P= .009).  CG: No significant change 50 (range, 34–68) and 51 (range, 34–75) in the first and third months; p = .657).  Accepting  IG: 1 month median 25 (18-27) 3 month 27 (17-31)  CG: 1 month median 25 (15-29) 3 month 25 (0-30)  Anxiety/concern  IG: 1 month median 10.50 (5-15) 3 month 11 (5-17)  CG: 1 month median 11 (6-15) 3 month 9 (5-16)  Social integration  IG: 1 month median 6 (4-12) 3 month 8 (4-13)  CG: 1 month median 7 (3-12) 3 month 6.50 (3-15)  Anger  IG: 1 month median 4 (1-6) 3 month 4 (1-6)  CG: 1 month median 2 (1-6) 3 month 2.50 (2-7) |
| Zhou et al. (2023) | OAI-20 | Baseline and 23º weeks | psychosocial adaptation score at discharge (baseline) (mean = 44.59, SD = 9.82) was lower than after the intervention (at the 23rd week after discharge or before temporary enterostomy reclosed (mean = 50.25, SD = 12.97) (P < 0.001).  positive emotion: Baseline mean 13.70 (3.50); 23 week mean = 14.94 (3.15),  negative emotion: Baseline mean 16.68 (7.48); 23 week mean = 19.76 (8.77),  social life adaptation Baseline mean 14.23 (2.98); 23 week mean 15.75 (3.37)  All were significantly higher after than before the intervention (P < 0.05) |
